# Circulating medium‐ and long‐chain acylcarnitines are associated with plasma P‐tau181 in cognitively normal older adults

**DOI:** 10.1111/jnc.16244

**Published:** 2024-10-30

**Authors:** Tahmida Sharmin, Pratishtha Chatterjee, James D. Doecke, Nicholas J. Ashton, Kevin Huynh, Steve Pedrini, Hamid R. Sohrabi, Benjamin Heng, Shaun Eslick, Henrik Zetterberg, Kaj Blennow, Manohar Garg, Ralph N. Martins

**Affiliations:** ^1^ Macquarie Medical School Macquarie University Macquarie Park New South Wales Australia; ^2^ Department of Pharmacy University of Rajshahi Rajshahi Bangladesh; ^3^ Florey Institute of Neuroscience and Mental Health University of Melbourne Melbourne Victoria Australia; ^4^ Australian eHealth Research Centre CSIRO Brisbane Queensland Australia; ^5^ School of Medical and Health Sciences Edith Cowan University Perth Western Australia Australia; ^6^ Department of Psychiatry and Neurochemistry University of Gothenburg Gothenburg Sweden; ^7^ Department of Old Age Psychiatry, Institute of Psychiatry, Psychology & Neuroscience King's College London London UK; ^8^ Metabolomics Laboratory Baker Heart and Diabetes Institute Melbourne Victoria Australia; ^9^ Alzheimer's Research Australia Nedlands Western Australia Australia; ^10^ School of Psychology Murdoch University Murdoch Western Australia Australia; ^11^ Clinical Neurochemistry Laboratory Sahlgrenska University Hospital Gothenburg Sweden; ^12^ Department of Neurodegenerative Disease UCL Institute of Neurology, Queen Square London UK; ^13^ UK Dementia Research Institute at UCL London UK; ^14^ Hong Kong Center for Neurodegenerative Diseases Clear Water Bay Hong Kong China; ^15^ Wisconsin Alzheimer's Disease Research Center, University of Wisconsin School of Medicine and Public Health University of Wisconsin‐Madison Madison Wisconsin USA

**Keywords:** acylcarnitines, Alzheimer's disease, biomarkers, cognitively normal older adults, initial pathogenesis, P‐tau181

## Abstract

Alzheimer's disease (AD) pathogenesis involves dysregulation in diverse biochemical processes. Nevertheless, plasma tau phosphorylated at threonine 181 (P‐tau181), a recognised AD biomarker, has been described to reflect early‐stage cortical amyloid‐β (Aβ) deposition in cognitively normal (CN) adults. Therefore, identifying changes in plasma metabolites associated with plasma P‐tau181 at the pre‐clinical stage may provide insights into underlying biochemical mechanisms to better understand initial AD pathogenesis. In the current study, plasma P‐tau181, quantified via single molecule array (Simoa) technology, and plasma metabolites, quantified via targeted‐mass spectrometry, were investigated for associations in CN older adults and upon stratification by positron emission tomography (PET)‐Aβ load. In addition, the P‐tau181‐linked metabolites were evaluated for cognitive performance and neuroimaging markers of AD and the potential to distinguish between CN Aβ− and CN Aβ+ individuals. Significant positive associations of medium‐ and long‐chain acylcarnitines (ACs) were observed with P‐tau181 in the entire cohort, CN Aβ− and CN Aβ+, suggesting a link between initial Aβ pathology and fatty acid oxidation‐mediated energy metabolism pathways. However, in CN Aβ−, additional linear associations of P‐tau181 were observed with muscle metabolism and nitric oxide homeostasis‐associated metabolites. Upon investigating the P‐tau181‐linked metabolites for cognitive performance, significant inverse correlations of the verbal and visual episodic memory and the global composite score were noted in CN Aβ+ with medium‐ and long‐chain ACs, suggesting prognostic value of ACs accompanying weaker cognitive performance. While investigating neuroimaging markers, ACs had positive associations with PET‐Aβ load and inverse associations with hippocampal volume in CN Aβ+, indicating connections of ACs with initial AD pathogenesis. Furthermore, based on receiver operating characteristics analysis, the associated ACs potentially classified PET‐Aβ status in older adults. Therefore, plasma P‐tau181‐linked circulating ACs may serve as potential prognostic markers for initial AD pathogenesis in CN older adults. However, further cross‐sectional and longitudinal research in highly characterised AD cohorts is needed to validate current findings.

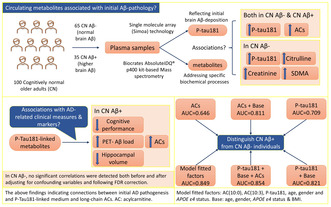

## Graphical abstract

Plasma tau phosphorylated at threonine 181 (P‐tau181), a recognised Alzheimer's disease (AD) biomarker, was reported to reflect early cortical Aβ deposition; however, the biochemical mechanisms associated with initial Aβ pathology remain to be investigated. The current study examined associations between P‐tau181 and plasma metabolites addressing specific biochemical processes, employing a cognitively normal (CN) older adult cohort and a targeted metabolomic platform. This study found positive relationships between P‐tau181 and ACs in both CN Aβ− and CN Aβ+ individuals—indicating a link between initial Aβ pathology and AC‐mediated energy metabolism pathways. Moreover, evaluation for clinical significance and categorising brain Aβ status further confirmed circulating ACs as markers for initial AD pathogenesis.

**Abbreviations**
Aβ: amyloid‐β
AA: Alzheimer's association
AC: acylcarnitine
AD: Alzheimer's disease
ADMA: asymmetric dimethylarginine
APOE: apolipoprotein E
ATP: adenosine triphosphate
AUC: area under the curve
BMI: body mass index
BP: blood pressure
CE: cholesteryl ester
CN: cognitively normal
CSF: cerebrospinal fluid
CV: coefficient of variation
DASS: Depression Anxiety and Stress Scale
DG: diglyceride
D‐KEFS: Delis–Kaplan executive function system
DNA: deoxyribonucleic acid
DSST: digit symbol substitution test
FBB: ^18^F‐florbetaben
FDR: false discovery rate
HRAM: high‐resolution accurate mass
KARVIAH: Kerr Anglican Retirement Village Initiative in Ageing Health
LLOD: lower limit of detection
LM: logical memory
LPC: lysophosphatidylcholine
MAC‐Q: Memory Complaint Questionnaire
MCI: mild cognitive impairment
Met‐SO: methionine sulphoxide
MMSE: mini‐mental state examination
MoCA: Montreal cognitive assessment
MRI: magnetic resonance imaging
NFTs: neurofibrillary tangles
NIA: National Institute on Aging
PC: phosphatidylcholine
PET: positron emission tomography
P‐tau181: tau phosphorylated at threonine 181
QC: quality control
RAVLT: Rey Auditory Verbal Learning Test
RCFT: Rey complex figure test
ROC: receiver operating characteristics
SDMA: symmetric dimethylarginine
Simoa: single molecule array
SM: sphingomyelin
SPSS: Statistical Package for the Social Sciences
SUVR: standard uptake value ratio
TCA: tricarboxylic acid cycle
TG: triglyceride
UHPLC–MS: ultra‐high‐performance liquid chromatography–mass spectrometry
WAIS: Wechsler Adult Intelligence Scale
WMS: Wechsler Memory Scale

## 1 INTRODUCTION

Given a multifactorial disorder, Alzheimer's disease (AD) pathogenesis involves dysregulation in diverse biochemical processes (Dorszewska et al., 2016; Koutsodendris et al., 2022; López‐Ortiz et al., 2021), including abnormal amyloid‐β (Aβ) production and impaired Aβ clearance, tau hyperphosphorylation, insufficiency in brain glucose utilisation, mitochondrial dysfunction, lipid dyshomeostasis, oxidative stress, neuroinflammation and neurotransmitter dyshomeostasis (Toledo et al., 2017; Wang et al., 2021; Wilkins & Trushina, 2018). This irreversible disorder has no cure and is characterised by a long (2–3 decades) pre‐clinical stage of neuropathological alterations and ultimate clinical manifestation with gradual memory loss and cognitive dysfunction leading to eventual death (Alzheimer's Association, 2022, 2023; Chatterjee et al., 2022). As such, early diagnosis at the pre‐clinical stage before considerable neuropathological alteration and subsequent early intervention to delay further progression is crucial. Therefore, low‐cost, less‐invasive, easily accessible and widely acceptable blood‐based biomarkers could be advantageous over the high‐cost, invasive and access‐limited neuroimaging and cerebrospinal fluid (CSF) biomarkers for community‐wide routine screening of AD.

Numerous studies (Chatterjee et al., 2022, 2023; Karikari et al., 2020, 2021; Simrén et al., 2021; Thijssen et al., 2020) have consistently reported plasma tau phosphorylated at threonine 181 (P‐tau181) as a potential and specific biomarker for (a) detecting AD pathology via distinguishing Aβ+ from Aβ− in cognitively normal (CN) adults as well as AD dementia from non‐AD dementias and (b) predicting clinical progression via forecasting gradual cognitive decline and disease development, that is, from CN Aβ+ to MCI or AD dementia. Furthermore, Shen et al. (2021) described plasma P‐tau181 as a predictive indicator of brain amyloidosis, tau pathology and glucose metabolism via positron emission tomography (PET) in a highly characterised AD cohort. Additionally, McGrath et al. (2022) demonstrated plasma P‐tau181 elevation in CN adults as an indicator of early multiple regions cortical Aβ depositions on brain PET. Nevertheless, studies (Gordon et al., 2016; Mattsson‐Carlgren et al., 2020) reported higher levels of CSF P‐tau181 accompanying the initial event of brain Aβ deposition with no detectable neurofibrillary tangles (NFTs) through tau radiotracer‐mediated PET imaging, indicating a higher P‐tau181 level in CSF reflecting a response to the growing brain Aβ load, instead of the mounting NFT suggested by Barthélemy et al. (2023). Therefore, P‐tau181 elevation is most likely a marker for initially growing Aβ pathology that precedes tau tangle pathology.

Metabolites are the low‐molecular‐weight molecules, intermediates or end products of cellular metabolic reactions, and specific metabolites provide essential insights into specific biological and pathophysiological processes and, thereby, are valuable to mirror underlying biochemical mechanisms (Belhaj et al., 2021; Kim et al., 2016; Pickford, 2019; Qiu et al., 2023; Wishart, 2019). Furthermore, previous metabolomic studies employed various analytical platforms and revealed several plasma metabolite alterations in AD individuals compared to controls or mild cognitive impairment (Lin et al., 2023; Ozaki et al., 2022; Wang et al., 2021; Wilkins & Trushina, 2018). In the current study, to explore initial biochemical mechanisms associated with AD pathogenesis, we investigated CN older adults for associations between plasma P‐tau181, an indicator of initial brain Aβ pathology, and plasma metabolites associated with energy homeostasis, mitochondrial dysfunction, insulin resistance, lipid disturbance, oxidative stress, nitric oxide homeostasis, neuroinflammation and neurotransmitter deregulation. Accordingly, the current study involved metabolomic analysis using the Biocrates Absolute*IDQ*® p400 kit (Chatterjee et al., 2021; Fritsche‐Guenther et al., 2022) – targeting plasma metabolites spanning 11 metabolite classes, including acylcarnitine (AC), lysophosphatidylcholine (LPC), phosphatidylcholine (PC), sphingomyelin (SM), ceramide, cholesteryl ester (CE), diglyceride (DG), triglyceride (TG), amino acid, biogenic amine and hexose, and following cross‐sectional investigation for associations with P‐tau181 in the entire cohort and separately in individuals with normal (CN Aβ−) and higher (CN Aβ+) PET‐Aβ build‐up. Furthermore, plasma P‐tau181‐linked metabolites were examined for associations with AD‐related clinical correlates, including cognitive performance, PET‐Aβ load and hippocampal volume. Moreover, P‐tau181‐linked metabolites were evaluated for the potential to predict PET‐Aβ status.

## 2 METHODS

### 2.1 Cohort participants

The participants were from the Kerr Anglican Retirement Village Initiative in Ageing Health (KARVIAH) cohort and were residents of Anglicare in New South Wales, Australia. One hundred thirty‐four (*n* = 134) participants of 200 initial volunteers met the inclusion and exclusion screening criteria for the KARVIAH study (Table 1). The Montreal Cognitive Assessment (MoCA) scores (between 18 and 25) were assessed by the study neuropsychologist on a case‐by‐case basis, following stratification of scores adjusting for age and education norms (Rossetti et al., 2011). One hundred and five participants underwent neuropsychological assessments, neuroimaging and blood sample collection, of which 100 were considered CN as they met the Mini‐Mental State Examination (MMSE) score ≥ 26 (Folstein et al., 1975) and were included in this study. All volunteers participated with prior informed consent, and the study has ethics approvals from the Bellberry Human Research Ethics Committee, Australia (ethics approval reference no. 2012‐09‐1086), and also the Macquarie University Human Research Ethics Committee (ethics approval reference no: 520231431450224). This study was not pre‐registered. The entire available cohort size that met the inclusion/exclusion screening criteria was utilised in this study.

**TABLE 1:** The inclusion and exclusion criteria for participant screening for the KARVIAH study.

KARVIAH cohort inclusion criteria	KARVIAH cohort exclusion criteria
An age range between 65 and 90 years	Previous dementia, as diagnosed accounting for the revised criteria, National Institute on Ageing (NIA)—Alzheimer's Association (AA) (McKhann et al., 2011)
Fluent in English	Presence of acute functional psychiatric disorder, including a lifetime history of schizophrenia or bipolar disorder
Adequate or corrected vision and hearing	History of stroke
Good general health	Depression, severe or extremely severe, based on the Depression Anxiety and Stress Scale (DASS)
No known significant cerebral vascular disease	Uncontrolled hypertension with systolic BP > 170 or diastolic BP > 100
No dementia or objective cognitive impairment with a Montreal Cognitive Assessment (MoCA) score ≥ 26	

### 2.2 Neuropsychological assessments

The cohort participants underwent a comprehensive battery of neuropsychological tests, including the (a) MoCA (Rossetti et al., 2011); (b) MMSE (Folstein et al., 1975); (c) Memory Complaint Questionnaire (MAC‐Q) (Crook et al., 1992); (d) Rey Auditory Verbal Learning Test – RAVLT List A, RAVLT short delay and RAVLT long delay (Estévez‐González et al., 2003); (e) Logical Memory – LM I and LM II (WMS‐III; Story A only) (Elwood, 1991); (f) Rey Complex Figure Test—RCFT 3 min and RCFT 30 min (Meyers & Meyers, 1995); (g) WAIS‐III Digit Span backward (Wechsler, 1987); (h) WAIS‐III Digit Symbol Substitution Test (DSST) (Wechsler, 1997); (i) Delis–Kaplan executive function system (D‐KEFS): category fluency (boys names) and category switching (fruits and furniture) tasks (Delis et al., 2001); (j) Controlled Oral Word Association Test (Patterson, 2011); (k) Stroop Test (Victoria version) (Strauss et al., 2006); (l) the Boston Naming Test (Saxton et al., 2000); (m) Wechsler Test of Adult Reading (Wechsler, 2001); and (n) the Depression Anxiety and Stress Scale (DASS) (Lovibond & Lovibond, 1995), previously reported in Chatterjee et al. (2018). The mean of the *z*‐score measures of specific neuropsychological tests was used to calculate composite scores to assess cognitive measures, including the verbal and visual episodic memory, the working memory and executive function and the global composite score (Table S1), as described in Chatterjee et al. (2018) and Chatterjee et al. (2021).

### 2.3 Blood collection, apolipoprotein E ( *APOE* ) genotyping and plasma P‐tau181 measurement

Blood collection involved a morning blood withdrawal from all participants after overnight fasting for at least 10 hours, followed by processing and storing blood samples at −80°C, as described by Goozee et al. (2018). *APOE* genotyping was performed utilising purified genomic DNA extracted from 0.5 mL whole blood (Goozee et al., 2018). For P‐tau181 measurement, plasma samples were quantified using an in‐house single molecule array (Simoa)‐based assay developed at the University of Gothenburg, Sweden (Ashton et al., 2021; Chatterjee et al., 2022; Karikari et al., 2020). A duplicate run was performed for calibrators, controls and samples for all assays. The lowest limit of quantification for analysis was 1 pg/mL, and the average coefficient of variation (CV) was 8%. In this cross‐sectional assessment, the total available sample was 97, with 65 CN Aβ− and 32 CN Aβ+.

### 2.4 Measurement of plasma metabolites

As per Chatterjee et al. (2021), a targeted analysis of endogenous plasma metabolite levels was done using the Biocrates Absolute*IDQ*® p400 kit employing an ultra‐high‐performance liquid chromatography‐mass spectrometry (UHPLC–MS, UHPLC—Thermo Scientific™ Vanquish™ and HRAM‐MS—Thermo Scientific™ Q Exactive Plus™) assay platform, in 2019. As specified in the manufacturer's protocol, all samples were deidentified, randomised and blinded before any analysis run. The Biocrates Absolute*IDQ*® p400 kit allows targeted analysis of up to 408 metabolite species; however, in this current study, metabolites with more than 40% of measurements under the lower limit of detection (LLOD) were excluded (Toledo et al., 2017), and thus, 216 metabolites were considered for further analysis. During sample analysis, a singlicate run was performed. For each plate run, a total of five quality control 2 (QC2), supplied by the manufacturers, were placed following every 19 to 20 test samples to confirm uniformity in quantitative performance and to normalise variations while considering intraplate and/or interplate, as described previously (Chatterjee et al., 2021).

### 2.5 Neuroimaging assessments

Within 3 months of blood sample collection, all participants underwent neuroimaging, including PET using ^18^F‐florbetaben (FBB) (Jovalekic et al., 2023; Sabri et al., 2015; Seibyl et al., 2016) for brain Aβ load and magnetic resonance imaging (MRI) for hippocampal volume, at Macquarie Medical Imaging in Sydney. FBB‐PET involved a 20 min static scanning (4 × 5 min dynamic frames) initiated at 50 min following intravenous injection of FBB. As stated by Bourgeat et al. (2015) and Zhou et al. (2014), the mean standard uptake value ratio, SUVR, of various neocortical regions, containing the (i) frontal, (ii) superior parietal, (iii) lateral temporal, (iv) lateral occipital and (v) anterior and posterior cingulate, were measured employing CapAIBL, an image processing software, to estimate the brain Aβ load. The participants were categorised based on their brain Aβ load with a cut‐off SUVR 1.35; SUVR < 1.35 was considered an average Aβ load, CN Aβ−, whereas SUVR ≥ 1.35 was considered a high brain Aβ load, CN Aβ+. Additionally, participants underwent an MRI utilising a General Electric 3Tesla scanner (Model 750W), previously described by Goozee et al. (2018). The acquired T1 MRI images were first normalised for the total intracranial volume, comprised of the grey matter, white matter and CSF, and then used to calculate hippocampal volumes.

### 2.6 Statistical analyses

Cohort characteristics were reported as mean ± SD (continuous variables) and percentage values (categorical variables). Independent sample *t*‐tests or chi‐square tests were used to compare demographic and clinical parameters between CN Aβ− and CN Aβ+ groups. Dependent variables were tested for normality using the Kolmogorov–Smirnov test and the Shapiro–Wilk test, and when required, a logarithmic or square root transformation was performed to approximate a better normal distribution. The entire available cohort size met the inclusion/exclusion screening criteria for the KARVIAH cohort and was previously used for similar studies. The entire cohort was utilised for the current study; therefore, no prior sample size calculation was performed, and no test for outliers was conducted. Correlations between plasma P‐tau181 and plasma metabolites were calculated using Spearman correlation coefficients (*ρ*) in all participants, CN Aβ− and CN Aβ+ groups. Additionally, linear models were utilised to calculate correlations after adjusting for confounding variables, including age, gender, *APOE* ε4 status and BMI, followed by false discovery rate (FDR) adjustment for correcting multiple comparisons; *p*‐values < 0.05 that survived FDR adjustment were considered significant. Furthermore, plasma metabolites associated with plasma P‐tau181 were investigated for associations with cognitive measures, PET‐Aβ load and hippocampal volume in all participants, CN Aβ− and CN Aβ+ groups utilising both Spearman correlation coefficients (*ρ*) and linear models following FDR corrections. Moreover, logistic regression model fitted values were used to construct the receiver‐operating characteristic (ROC) curves, thereby calculating the areas under the curves (AUCs). Statistical analyses were conducted using the statistical software IBM SPSS (v 28), GraphPad Prism (v 9.2.0) and R statistical environment (v 4.1.0); however, graphs were plotted for visualisation using either GraphPad Prism (v 9.2.0) or R statistical environment (v 4.1.0).

## 3 RESULTS

### 3.1 Cohort characteristics

The cohort participant characteristics, including demographic, cognitive measures, hippocampal volume, PET‐Aβ Load and plasma P‐tau181 measures, are presented in Table 2. As the demographic factors reported previously (Chatterjee et al., 2021), the cohort differed significantly between the CN Aβ− and CN Aβ+ groups for the *APOE* ε4 carrier status; however, no statistically significant differences were noted for age, gender, body mass index (BMI) and education status. PET‐Aβ load (*t*‐test df = 98, *t* = −15.693, *p*‐value <0.0001) and AD‐related plasma biomarker, P‐tau181 (*t*‐test df = 95, *t* = −3.367, *p*‐value = 0.001), differed significantly between the CN Aβ− and CN Aβ+ individuals. No significant differences were found between the study groups regarding cognitive measures and hippocampal volume.

**TABLE 2:** Cohort characteristics including demographic, cognitive measures, brain imaging and plasma measures of study participants.

Characteristics	Number of participants (*N*)	Cognitively normal (CN) individuals stratified by PET‐Aβ load		*p*‐value
		CN Aβ− (*n* = 65)	CN Aβ+ (*n* = 35)	
Age (years)	100	77.615 ± 5.556	79.228 ± 5.380	0.165
Female (*n* (%))	100	46 (70.769)	22 (62.857)	0.419
*APOE* ε4 carrier (*n* (%))	100	5 (7.692)	16 (45.714)	**<0.0001**
Body Mass Index (BMI) (kg/m^2^)	100	27.385 ± 4.477	28.054 ± 4.735	0.486
Education (years)	100	14.846 ± 3.373	13.643 ± 2.919	0.078
MMSE	100	28.508 ± 1.161	28.800 ± 1.106	0.225
Verbal and visual episodic memory (*z* score)	100	0.087 ± 0.601	−0.162 ± 0.821	0.085
Working memory and executive function (*z* score)	100	0.079 ± 0.622	−0.148 ± 0.661	0.092
Global composite (*z* score)	100	0.070 ± 0.488	−0.130 ± 0.625	0.080
Hippocampal volume left %	96 (64, 32)	0.195 ± 0.020	0.194 ± 0.019	0.805
Hippocampal volume right %	96 (64, 32)	0.199 ± 0.021	0.199 ± 0.018	0.891
FBB‐PET SUVR	100	1.158 ± 0.086	1.714 ± 0.261	**<0.0001**
Plasma P‐tau181 level (pg/mL)	97 (65, 32)	13.549 ± 5.693	17.273 ± 5.578	**0.001** ^a^

*Note*: Categorical measures are presented as counts and percentages, whereas continuous measures are presented as mean ± SD. Independent sample *t*‐tests or chi‐square tests were used to compare the measures between the CN Aβ− and CN Aβ+ groups. All values are presented up to three decimal places for the complete table, except for the <0.0001 values, and p‐values < 0.05 were considered statistically significant (bold font). PET‐Aβ load was categorised based on the FBB‐PET SUVR cut‐off 1.35. *n* represents the count.

Abbreviations: APOE, apolipoprotein E; FBB‐PET, ^18^F‐florbetaben positron emission tomography; MMSE, min‐mental state examination; SUVR, standardised uptake value ratio.

^a^ The *p*‐value obtained following logarithmic transformation to meet the requirement for normal distribution.

### 3.2 Associations of AD‐related genetic and demographic factors with plasma metabolites

All 216 metabolites were assessed for normal distribution, and as appropriate, a logarithmic or square root transformation was performed to approximate better normality and then evaluated for associations with AD‐related confounding variables. While considering the *APOE* ε4 carrier status, none of the plasma metabolites differed significantly between the *APOE* ε4− and *APOE* ε4+ individuals, except for DG(44:3), which showed higher plasma levels in *APOE* ε4+ (mean ± SD: 3.07 ± 1.74 μM, *n* = 21) compared to ε4− (mean ± SD: 2.23 ± 1.51 μM, *n* = 79) with *p*‐value 0.034 (Figure S1), consistent with Chatterjee et al. (2021). Upon looking at demographic factors, significant associations were observed with age, gender and BMI in the entire cohort. Specifically, eight AC species [AC(3:0), AC(8:1), AC(10:0), AC(10:2), AC(10:3), AC(12:0), AC(12:1) and AC(14:1)], LPC(17:1), two amino acids (citrulline and proline) and three biogenic amines [creatinine, methionine sulphoxide (Met‐SO) and symmetric dimethylarginine (SDMA)] were found to have positive associations with age, that is, the higher the age, the higher was the plasma levels of these metabolites (Table S2). In contrast, negative associations were observed between age and 13 PCs, 7 SMs, CE(18:2), TG(56:7) and amino acid tryptophan. Plasma levels of ACs [AC(3:0), AC(10:3), AC(16:0), AC(18:0), AC(18:1) and AC(18:2)], LPCs [LPC(17:1), LPC(18:2), LPC(20:4) and LPC‐O(18:1)], amino acids (aspartate, glutamate, isoleucine, leucine, methionine, proline and valine) and biogenic amine creatinine were significantly lower in females compared to males (Table S3). In contrast, plasma levels were significantly higher in female participants for PCs, SMs, CEs, two DGs, TG(54:2) and amino acid glycine. Furthermore, significant positive correlations of BMI were observed with PC(44:1), three SMs, hexoses, two CEs, five DGs, five TGs, five amino acids (alanine, glutamate, proline, tyrosine and valine) and two biogenic amines [kynurenine and asymmetric dimethylarginine (ADMA)] (Table S4). In contrast, inverse associations were found with PC(39:5), PC(42:6), PC‐O(36:2) and CE(19:2).

### 3.3 Plasma metabolites associated with plasma P‐tau181

**FIGURE 1:**
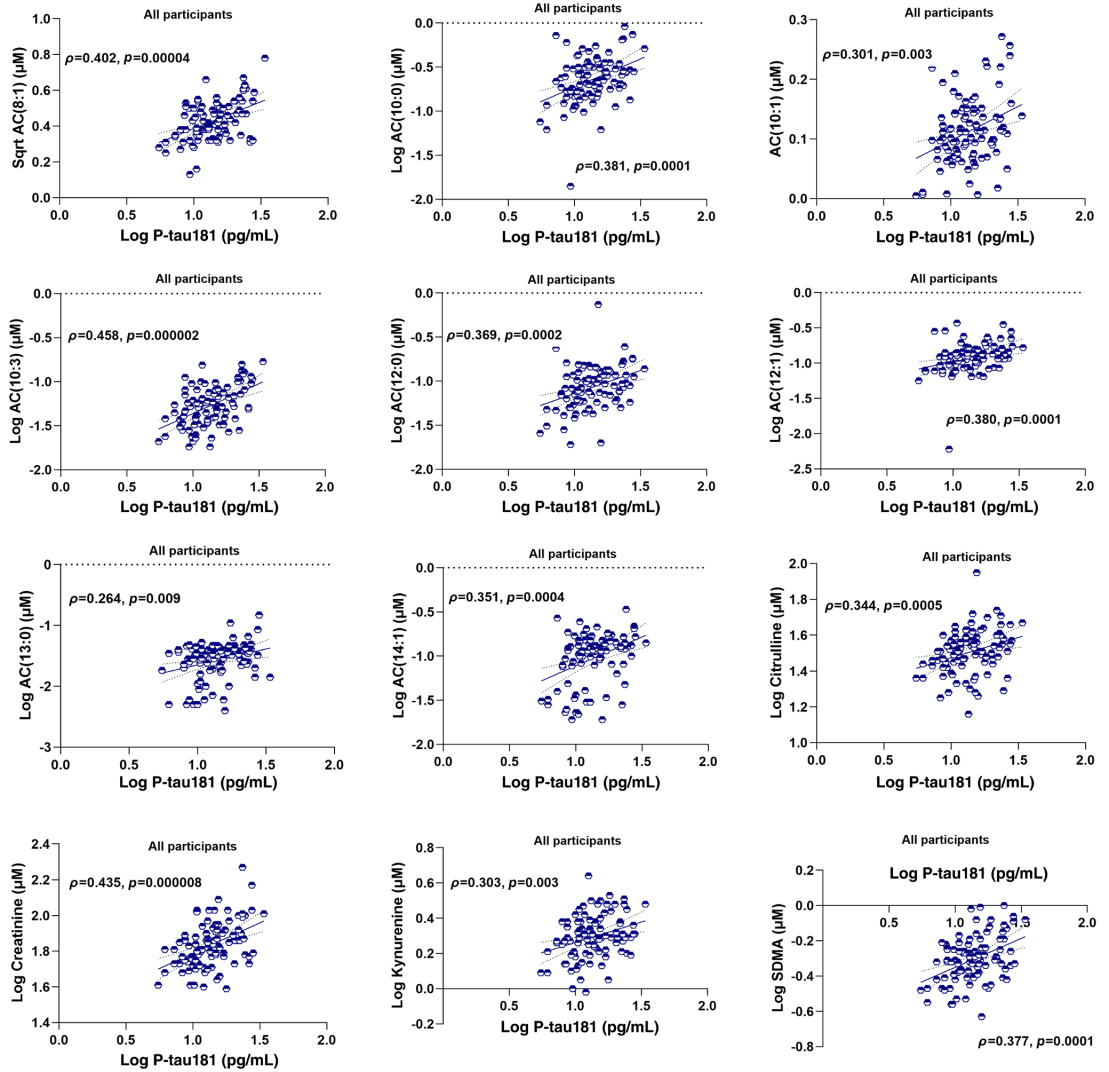
Plasma metabolites associated with plasma P‐tau181 in all participants. Metabolites significantly correlating with P‐tau181 both before and after adjusting for confounding variables and survived false discovery rate correction have been reported. Of 216 metabolites, only 12 metabolites belonging to three metabolite classes, including acylcarnitines (ACs), 1 amino acid and 3 biogenic amines, were found to have significant positive correlations with P‐tau181 in all participants.

**FIGURE 2:**
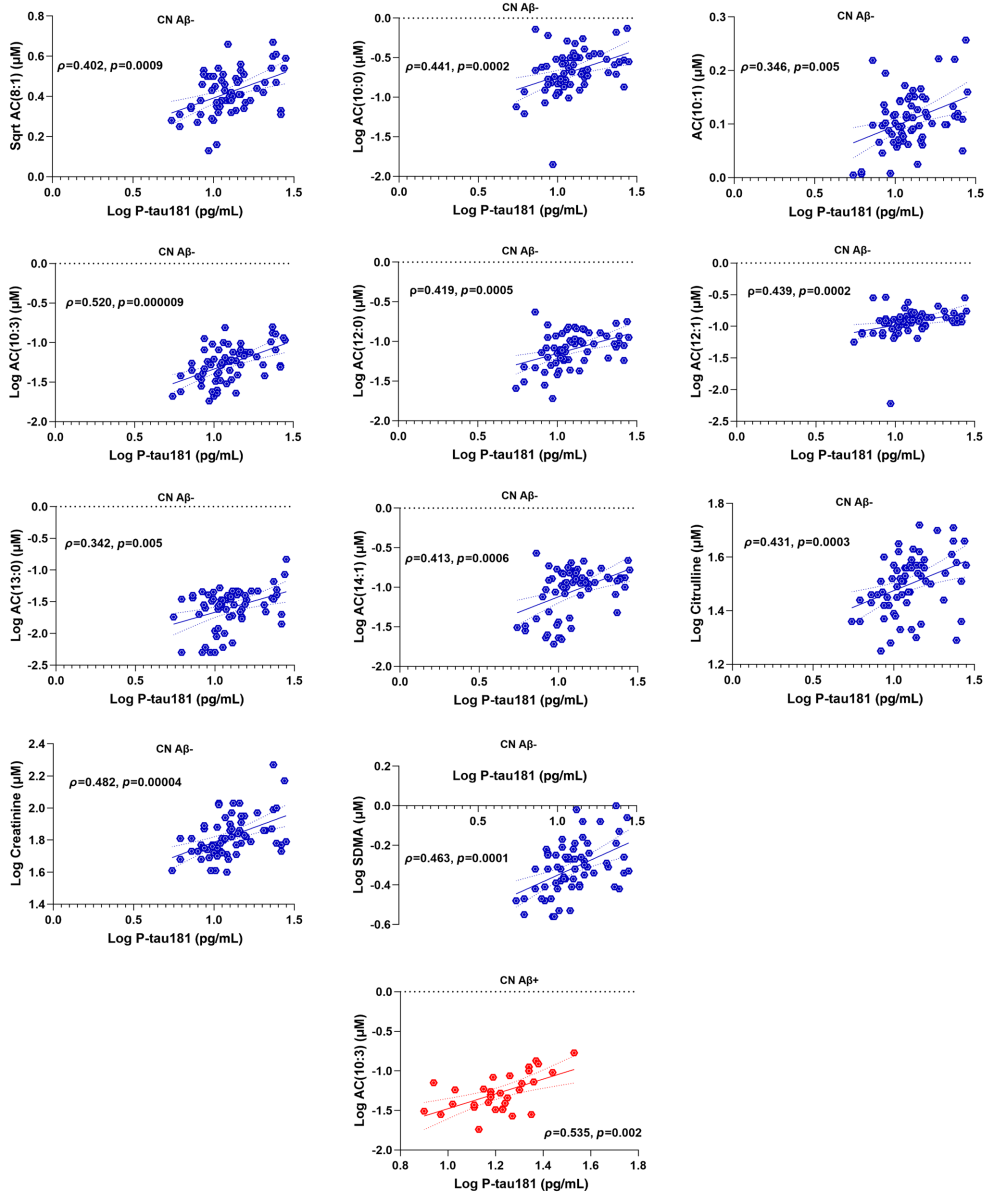
Plasma metabolites associated with plasma P‐tau181 separately in CN Aβ− (blue) and CN Aβ+ (red). Metabolites significantly correlating with P‐tau181 both before and after adjusting for confounding variables and survived false discovery rate correction have been reported. Only acylcarnitines (AC) had significant positive correlations with P‐tau181 both in CN Aβ− and CN Aβ+ individuals. However, in CN Aβ−, additional positive correlations of P‐tau181 were observed with creatinine, SDMA and citrulline.

Metabolites associated with P‐tau181 were investigated in all participants and separately in CN Aβ− and CN Aβ+ utilising the Spearman correlation coefficient and then generalised linear model accounting for age, gender, *APOE* ε4 status and BMI following FDR adjustment for correcting multiple comparisons (Table 3; Figures 1 and 2). In all participants, significant positive correlations of P‐tau181 were observed with AC(8:1), AC(10:0), AC(10:1), AC(10:3), AC(12:0), AC(12:1), AC(13:0), AC(14:1), creatinine, kynurenine, SDMA and citrulline both before and after adjusting for covariates and following FDR correction (Table 3; Figure 1). While investigated separately in CN Aβ− and CN Aβ+ individuals, significant positive correlations of P‐tau181 were observed with ACs both before and after controlling for confounding variables and following FDR adjustment (Table 3; Figure 2). However, in CN Aβ−, additional positive correlations of P‐tau181 were observed with creatinine, SDMA and citrulline, which survived controlling for confounding variables and following FDR adjustment (Table 3; Figure 2).

**TABLE 3:** Plasma metabolites associated with plasma P‐tau181 in all participants, CN Aβ− and CN Aβ+.

	All participants (*n* = 97)				CN Aβ− (*n* = 65)				CN Aβ+ (*n* = 32)			
	*ρ*	*p*‐value	*β*	*p*‐value	*ρ*	*p*‐value	*β*	*p*‐value	*ρ*	*p*‐value	*β*	*p*‐value
Lipids												
AC(8:1)^Ϯ^	0.402	**0.00004**	0.571	**0.00002** ^‡^	0.402	**0.0009**	0.586	**0.0002** ^‡^	0.446	**0.010**	0.532	**0.010**
AC(10:0)^$^	0.381	**0.0001**	0.205	**0.0001** ^‡^	0.441	**0.0002**	0.210	**0.001** ^‡^	0.335	0.061	0.191	**0.029**
AC(10:1)	0.301	**0.003**	0.925	**0.0004** ^‡^	0.346	**0.005**	1.020	**0.003** ^‡^	0.217	0.232	0.507	0.165
AC(10:2)^$^	0.258	**0.011**	0.075	0.054	0.255	**0.040**	0.080	0.074	0.339	0.058	0.019	0.772
AC(10:3)^$^	0.458	**0.000002**	0.302	**0.000002** ^‡^	0.520	**0.000009**	0.311	**0.00007** ^‡^	0.535	**0.002**	0.274	**0.003** ^‡^
AC(12:0)^$^	0.369	**0.0002**	0.200	**0.002** ^‡^	0.419	**0.0005**	0.233	**0.006** ^‡^	0.308	0.086	0.093	0.307
AC(12:1)^$^	0.380	**0.0001**	0.209	**0.003** ^‡^	0.439	**0.0002**	0.229	**0.005** ^‡^	0.298	0.097	0.189	0.119
AC(13:0)^$^	0.264	**0.009**	0.159	**0.001** ^‡^	0.342	**0.005**	0.204	**0.0001** ^‡^	0.039	0.830	0.012	0.895
AC(14:1)^$^	0.351	**0.0004**	0.172	**0.001** ^‡^	0.413	**0.0006**	0.175	**0.004** ^‡^	0.308	0.086	0.115	0.182
AC(16:0)^Ϯ^	0.208	**0.042**	0.527	0.118	0.246	0.050	0.209	0.622	0.263	0.146	0.792	0.083
AC(18:1)^Ϯ^	0.243	**0.016**	0.466	0.080	0.282	**0.023**	0.355	0.269	0.209	0.250	0.803	**0.036**
LPC(17:1)	0.242	**0.017**	0.366	0.170	0.145	0.248	−0.034	0.920	0.392	**0.026**	0.812	**0.038**
TG(56:7)^$^	−0.233	**0.022**	−0.110	0.183	−0.220	0.078	−0.085	0.384	−0.168	0.357	−0.225	0.113
Amino acids												
Citrulline^$^	0.344	**0.0005**	0.297	**0.015** ^‡^	0.431	**0.0003**	0.474	**0.003** ^‡^	0.238	0.189	0.082	0.593
Proline^$^	0.258	**0.011**	0.179	0.127	0.227	0.069	0.092	0.578	0.391	**0.027**	0.290	**0.045**
Biogenic amines												
Creatinine^$^	0.435	**0.000008**	0.482	**0.0002** ^‡^	0.482	**0.00004**	0.555	**0.001** ^‡^	0.430	**0.014**	0.241	0.245
Kynurenine^$^	0.303	**0.003**	0.317	**0.009** ^‡^	0.368	**0.003**	0.315	**0.030**	0.267	0.139	0.180	0.393
SDMA^$^	0.377	**0.0001**	0.391	**0.001** ^‡^	0.463	**0.0001**	0.490	**0.001** ^‡^	0.306	0.088	0.148	0.413

*Note*: Plasma metabolites correlated with plasma P‐tau181 were investigated using the Spearman correlation, and *p*‐values < 0.05 were considered significant. Generalised linear models were utilised to explore plasma metabolites associated with plasma P‐tau181 upon adjusting for age, gender, *APOE* ε4 status and BMI and *p*‐values < 0.05 that survived FDR adjustment (denoted as ‡) were considered significant. ‘*n*’ represents the number of participants, ‘*ρ*’ represents the Spearman correlation coefficient and ‘*β*’ represents the beta coefficient. ^Ϯ^ Represents square root transformation and ^$^ represents logarithmic transformation to meet the requirement for normal distribution. AC, acylcarnitine; LPC, lysophosphatidylcholine; TG, triglyceride; SDMA, symmetric dimethylarginine. Plasma metabolites significantly correlating (*p*‐values < 0.05, bold) with P‐tau181 both before and after adjusting for confounding variables and survived FDA correction (*p*‐values < 0.05, bold and ‘^‡^’ signed) were considered statistically significant.

### 3.4 Associations of cognitive measures, PET‐Aβ load and hippocampal volume with P‐tau181‐linked metabolites

**FIGURE 3:**
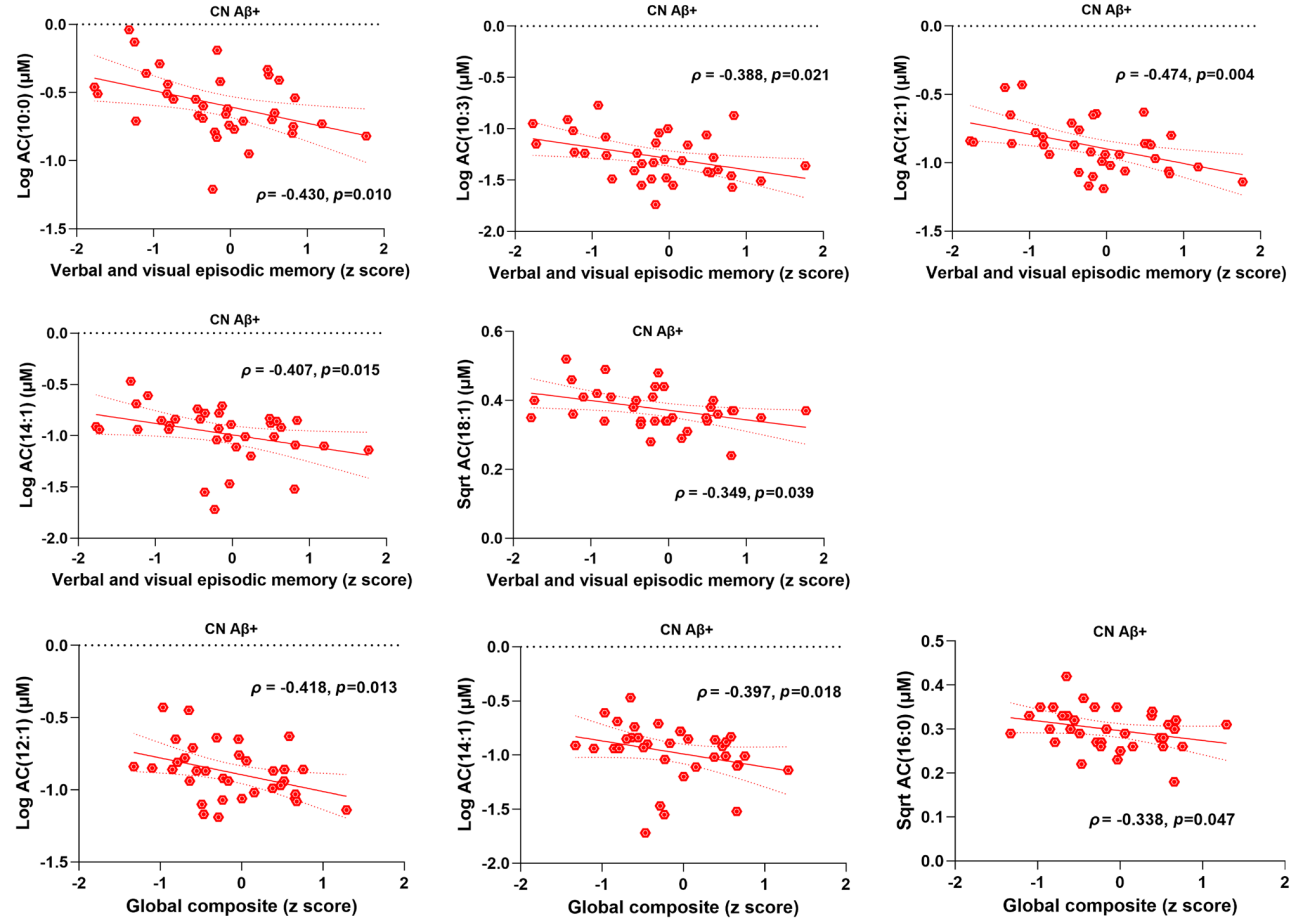
Correlations of cognitive performance with P‐tau181‐linked metabolites. Upon investigation, only acylcarnitine (AC) species had significant inverse correlations with cognitive measures, namely verbal and visual episodic memory and the global composite score, both before and after controlling for confounding variables and following false discovery rate (FDR) adjustment for correcting multiple comparisons. In CN Aβ−, no significant correlations with cognitive measures were detected both before and after controlling for confounding variables and following FDR adjustment.

Three different cognitive measures, namely the verbal and visual episodic memory, the working memory and executive function and the global composite score, were investigated to evaluate the cognitive performance of the cohort participants. Statistically significant negative associations of verbal and visual episodic memory were observed with AC(10:0), AC(10:3), AC(12:1), AC(14:1) and AC(18:1) in CN Aβ+ both before and after adjusting for confounding variables and following FDR correction (Figure 3; Table S5). As presented in Table S6, no significant correlations with working memory and executive function were detected both before and after adjustment for confounding variables and subsequent FDR correction in any of the study groups. While considering associations with the global composite score, the statistically significant inverse correlations both before and after adjusting for confounding variables and following FDR correction were with AC(12:1), AC(14:1) and AC(16:0) only in CN Aβ+ (Figure 3; Table S7).

**FIGURE 4:**
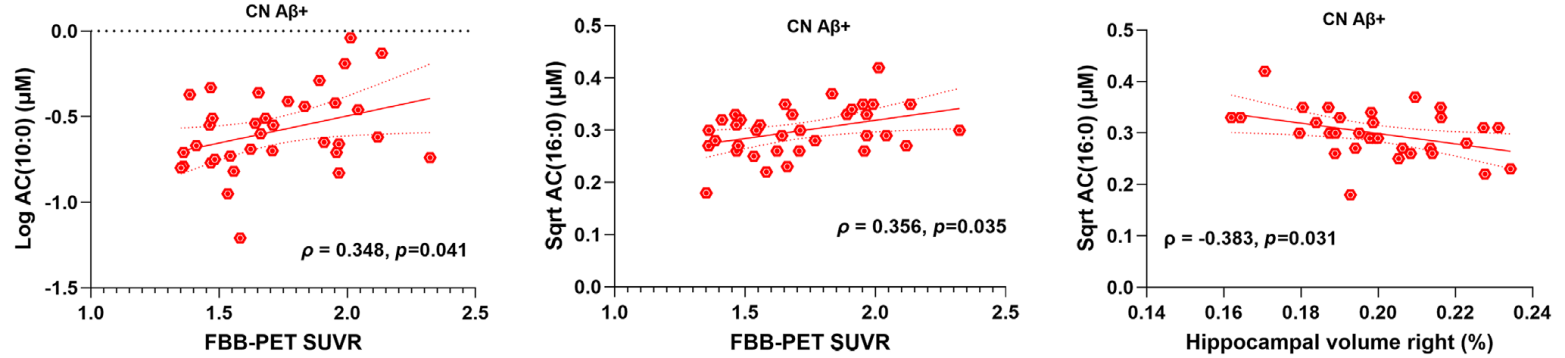
Correlations of neuroimaging biomarkers with P‐tau181‐linked metabolites. Upon investigation, only acylcarnitine (AC) species were found to have significant positive correlations with AD‐related neuroimaging biomarkers, including PET‐Aβ load and hippocampal volume, only in CN Aβ+ both before and after controlling for confounding variables and following false discovery rate (FDR) adjustment for correcting multiple comparisons. In CN Aβ−, no significant correlations with neuroimaging biomarkers were detected both before and after controlling for confounding variables and following FDR adjustment.

Furthermore, upon investigating associations with the PET‐Aβ load, AC(10:0) and AC(16:0) had significant positive associations while considering bivariate correlation; additionally, the statistical significance sustained upon linear model testing and following multiple corrections only in CN Aβ+ (Figure 4; Table S8). Furthermore, while studying hippocampal volume, the only Spearman correlation that persisted statistical significance after controlling for confounding variables and survived FDR adjustment was between AC(16:0) and hippocampal volume of the right hemisphere (Figure 4; Tables S9 and S10).

### 3.5 Evaluation of the associated ACs as a predictor of brain Aβ status in CN older adults

**FIGURE 5:**
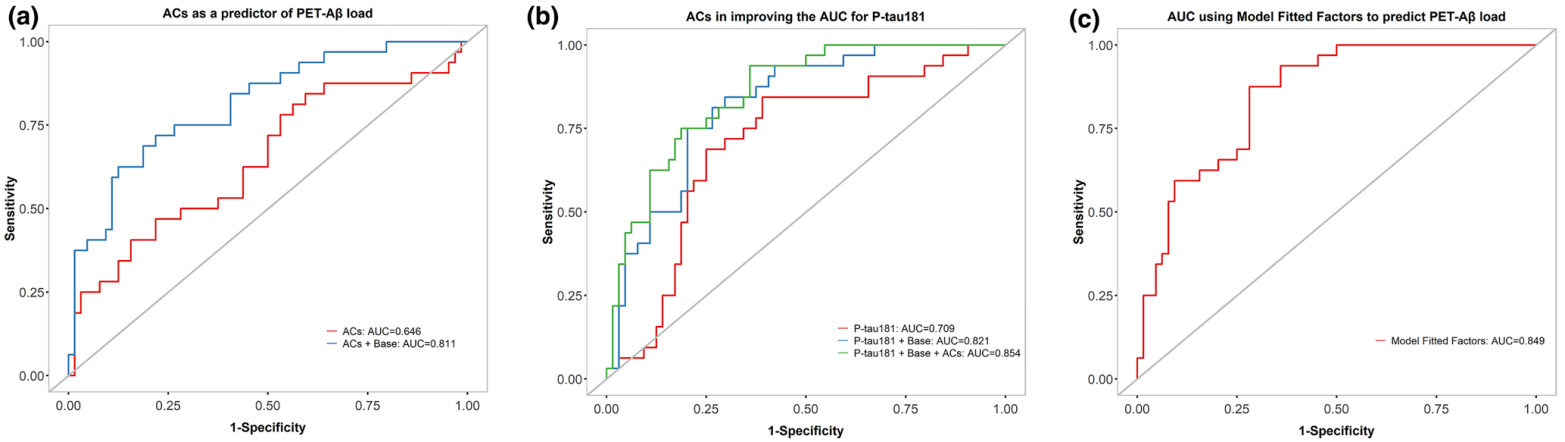
Receiver operating characteristic (ROC) curves to classify brain Aβ status in cognitively normal older adults (*n* = 96) for (a) associated ACs, including AC(10:0), AC(10:3), AC(12:1), AC(14:1), AC(16:0) and AC(18:1) and ACs + Base; (b) P‐tau181, P‐tau181 + Base and P‐tau181 + Base + Acs; and (c) model fitted factors, including AC(10:0), AC(10:3), P‐tau181, age, gender and *APOE* ε4 status. AUC, area under the curve; Base comprises age, gender, *APOE* ε4 status and BMI.

The predictive potential of the associated ACs, including AC(10:0), AC(10:3), AC(12:1), AC(14:1), AC(16:0) and AC(18:1), to classify the brain Aβ status in CN older adults was investigated using ROC curves (Figure 5). The associated ACs potentially categorised the brain Aβ status alone (AUC = 64.6%) and with the base model (comprising AD risk factors such as age, gender, *APOE* ε4 status and BMI, AUC = 81.1%) in CN older adults (Figure 5a). Moreover, using a linear combination of these ACs with P‐tau181 and base potentially classified the brain Aβ status with an AUC of 85.4% (Figure 5b), which was higher than the AUC predicted by P‐tau181 alone (AUC = 70.9%, *n* = 96, DeLong's test *p*‐value = 0.012) and P‐tau181 + Base (AUC = 82.1%, *n* = 96, DeLong's test *p*‐value = 0.270).

However, given the small sample size (*n* = 96) and to avoid model overfitting, a multivariate model fitting procedure (stepAIC—stepwise logistic regression) was performed to keep only those predictive factors that can explain variance in the brain amyloid status. Using this model selection procedure, only two ACs were chosen, AC(10:0) and AC(10:3), along with P‐tau181, age, gender and *APOE* ε4 status. The resulting AUC from this model was 84.9% (Figure 5c), which was significantly greater than P‐tau181 alone (DeLong's test *p* value = 0.014) in distinguishing CN Aβ+ from CN Aβ− individuals.

## 4 DISCUSSION

This research, to the best of our knowledge, is the first to describe plasma metabolites and allied biochemical pathways associated with AD‐related initial Aβ pathology, as assessed by the biomarker P‐tau181, in CN older adults. In this study, significant positive correlations of circulating medium‐ and long‐chain ACs were found with P‐tau181 in the entire cohort and separately in CN Aβ+ and CN Aβ− individuals both before and after adjusting for confounding variables and subsequent FDR corrections—reflecting a link between initial Aβ pathology and AC‐mediated energy metabolism pathways in CN older adults. Interestingly, previous studies (Cristofano et al., 2016; Mapstone et al., 2014) reported lower levels of some blood ACs in MCI and AD individuals compared to healthy controls. Therefore, the linear associations observed between P‐tau181 and ACs in this study may likely reflect initial compensatory mechanisms to the early neuropathological alterations in CN older adults. However, in CN Aβ− individuals, additional correlations of P‐tau181 were observed with three other metabolites, creatinine, citrulline and SDMA, associated with muscle metabolism and nitric oxide homeostasis.

Furthermore, upon investigating the P‐tau181‐linked metabolites for associations with cognitive performance, significant negative correlations of the verbal and visual episodic memory were observed with AC(10:0), AC(10:3), AC(12:1), AC(14:1) and AC(18:1) in the CN Aβ+ individuals. Therefore, the weaker the verbal and visual episodic memory performance, the higher the plasma medium‐ and long‐chain ACs in CN Aβ+ individuals. Similar negative correlations of the global composite scores were detected with AC(12:1), AC(14:1) and AC(16:0) in CN Aβ+ individuals both before and after adjusting for confounding variables and subsequent FDR correction. While absent in the CN Aβ− group, these findings indicate an inverse relationship between the cognitive measures and circulating medium‐ and long‐chain ACs in pre‐clinical AD, suggesting the predictive value of circulating ACs accompanying weaker cognitive performance.

Additionally, upon examining the P‐tau181‐linked metabolites for associations with neuroimaging biomarkers, significant correlations of AC species were spotted only in CN Aβ+ individuals; AC(10:0) and AC(16:0) were found to have positive correlations with PET‐Aβ load, and AC(16:0) had an inverse correlation with hippocampal volume both before and after controlling for confounding variables and subsequent FDR adjustment. Therefore, a trend of higher brain Aβ deposition and lower hippocampal volume was observed with a higher plasma level of AC(16:0), indicating associations of initial AD pathogenesis with AC‐associated pathways in the early stage of pre‐clinical AD. Moreover, while evaluating the potential to differentiate CN Aβ+ from CN Aβ− individuals, the associated medium‐ and long‐chain ACs potentially classified PET‐Aβ status and, in combination with P‐tau181 and AD risk factors, performed significantly better than the AUC for P‐tau181.

Acylcarnitines are the metabolic intermediates of fatty acid oxidation and are essentially generated and utilised in numerous cellular energy metabolism pathways (Dambrova et al., 2022). The mitochondria are the central organelles that contribute to most AC synthesis; additionally, peroxisomal metabolisms contribute to some extent to AC production (Hunt et al., 2012). Furthermore, as described in previous studies (Rinaldo et al., 2008; Schooneman et al., 2013), fatty acid oxidation is the primary source of most AC productions; however, other sources that contribute to AC synthesis include glucose, some amino acids (Rinaldo et al., 2008) and ketone bodies (Soeters et al., 2012). While circulating, medium‐ and long‐chain ACs are entirely obtained from fatty acid metabolism, and short‐chain ACs are generated from amino acids, glucose and fatty acid degradation (Makrecka‐Kuka et al., 2017). Nevertheless, fasting or high energy demand increases AC concentrations because of increased rates of fatty acid oxidation (Liepinsh et al., 2014; Makrecka et al., 2014, as cited in Makrecka‐Kuka et al., 2017).

The well‐established function of ACs is to help transport long‐chain fatty acids from the cytosol into the mitochondrial matrix, wherein the fatty acid undergoes a series of steps for β‐oxidation—producing adenosine triphosphates (ATPs) and acetyl‐coenzyme As (acetyl‐CoAs). The generated acetyl‐CoAs undergo either complete oxidation for energy production (ATPs) through the citric acid cycle or ketone body synthesis in the liver if the body needs to form ketones during starvation or to fuel the brain. During fasting, energy homeostasis is maintained via shifting to alternative energy substrate utilisation; predominantly fatty acid oxidation in the liver, cardiac muscle and skeletal muscle. Generally, the brain utilises ketone bodies produced in the liver following fatty acids β‐oxidation; additionally, the heart and skeletal muscle can use ketone bodies as glucose‐sparing substrates (Costa et al., 1999; Houten & Wanders, 2010; Longo et al., 2006, 2016). Besides, studies have reported ACs for essential functions in the brain, including energy metabolism (Aureli et al., 1990, 1994; Pettegrew et al., 2000; Rosenthal et al., 1992; Scafidi et al., 2010), neurotransmitter production (Ferreira & McKenna, 2017; Pettegrew et al., 2000; Scafidi et al., 2010) and neuroprotection (Ferreira & McKenna, 2017; Hiskens et al., 2023; Jones et al., 2010; Pettegrew et al., 2000; Scafidi et al., 2010). Furthermore, AC(16:0) is essential for synthesising complex lipids associated with brain plasticity, neural membranes and signal transduction, as per Jones et al. (2010).

**FIGURE 6:**
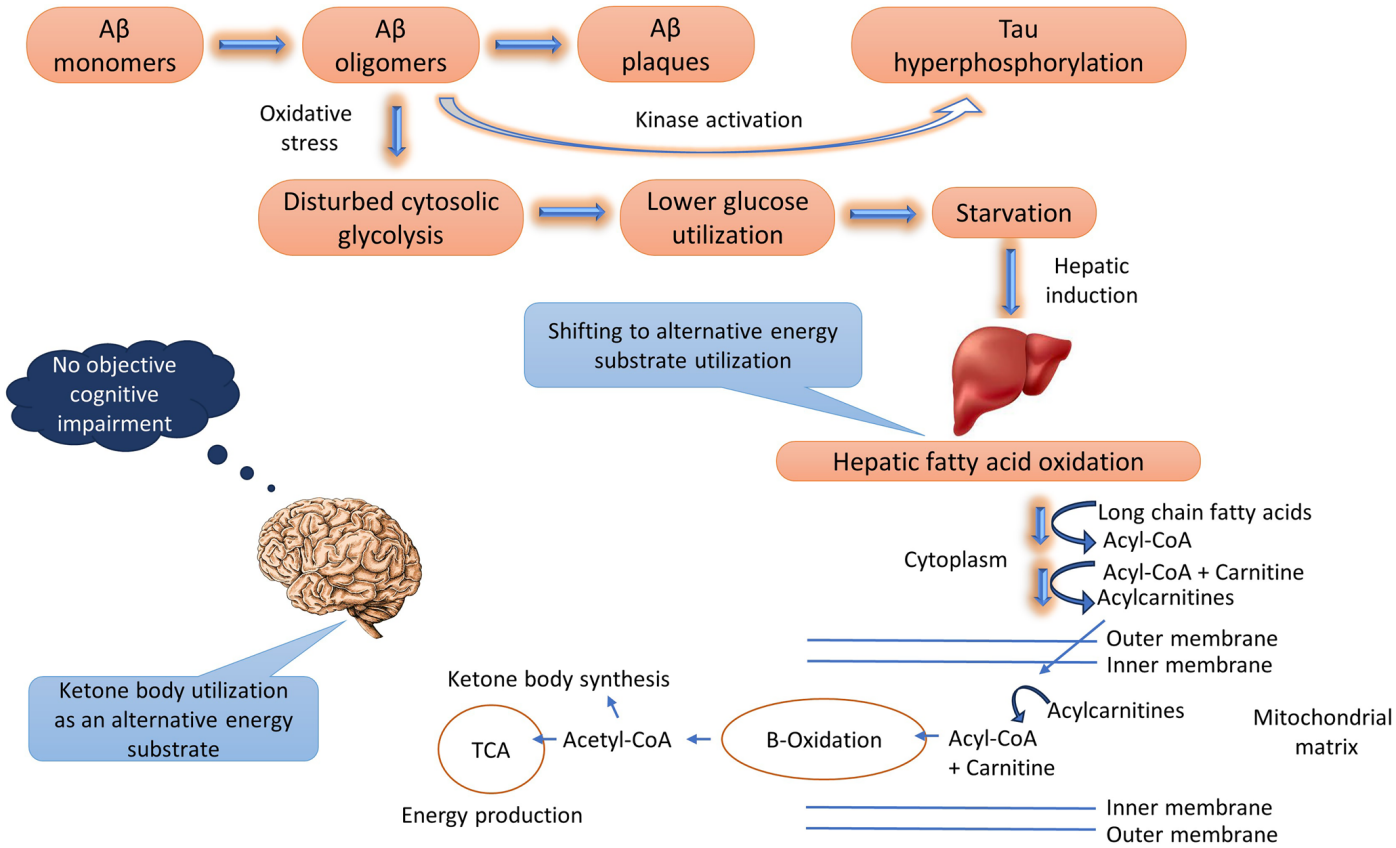
The proposed association mechanism between initial Aβ pathology and acylcarnitine‐mediated energy metabolism pathways in cognitively normal older adults. The initial Aβ pathology‐mediated starvation induces hepatic fatty acid β‐oxidation through intermediary production of acylcarnitines, leading to the eventual formation of acetyl‐CoAs, which then undergo energy production through the TCA cycle or hepatic ketone body synthesis. TCA, tricarboxylic acid cycle.

However, many pre‐clinical AD studies demonstrated Aβ pathology concurrently with reduced glucose metabolism as initial markers of AD brain (Butterfield & Halliwell, 2019; Caminiti et al., 2018; Gordon et al., 2018). Furthermore, Malkov et al. (2021) reported initial Aβ oligomer‐mediated oxidative stress and subsequent cytosolic glycolysis disruption as an onset mechanism for brain hypometabolism. Additionally, McGrath et al. (2022) have recently reported plasma P‐tau181 in community doweling CN individuals as a potential marker of initial brain amyloidosis rather than reflecting vascular risk factors. Therefore, taken together with the previous reports, the most likely mechanism contributing to current findings could be that the oxidative stress caused by initial Aβ pathology induces disturbance in cytosolic glycolysis, leading to lower‐glucose utilisation by the brain and subsequent starvation. This starvation, in turn, stimulates hepatic fatty acid metabolism as a compensatory mechanism for shifting to alternative energy substrate utilisation, leading to the production of metabolic intermediates, that is, medium‐ and long‐chain ACs, which subsequently undergo fatty acid β oxidation and successive acetyl‐CoAs production. These acetyl‐CoAs then undergo energy production through the TCA cycle or go through the hepatic synthesis of ketone bodies, which the body utilises during fasting or starvation to fuel the brain (Figure 6). Furthermore, in line with these findings, Dhitavat et al. (2002) demonstrated an in vitro study of the beneficial effects of ACs in retarding Aβ‐induced oxidative stress and following cell death, further confirming the protective effects of ACs through mitigating oxidative stress and upholding ATP levels.

The current study on investigating metabolites linked with P‐tau181 and subsequent investigation of P‐tau181‐linked metabolites for AD‐related clinical significance, as well as their evaluation for the potential to classify PET‐Aβ load, has many strengths. Firstly, this study utilised a high‐resolution mass spectrometry‐based BIOCRATES Absolute*IDQ®* p400HR kit to quantify plasma metabolites. Secondly, plasma P‐tau181 was quantified using ultra‐sensitive (Simoa) technology. Thirdly, all investigated P‐tau181‐linked metabolites were evaluated for association with the core AD‐related clinical measures and markers, including cognitive performance and neuroimaging biomarkers. Finally, all statistical assessments were considered upon adjusting for AD risk factors such as age, gender, BMI and *APOE* ε4 status, and subsequent FDR adjustment for multiple comparisons. Nevertheless, this study has many limitations, including the study nature being cross‐sectional and pre‐symptomatic, using a modest sample size, the restraint of parallel study using both CSF and Plasma P‐tau181, the restraint of accounting for the plasma levels of ketone bodies, medication and/or diet used and the limitation of adjusting for multiple races and ethnic backgrounds.

In conclusion, this research demonstrated circulating ACs associated with P‐tau181, revealing valuable insights into AC‐associated energy metabolism pathways accompanying the initial Aβ pathology in CN older adults. Furthermore, this study disclosed medium‐ and long‐chain plasma ACs that are associated with cognitive performance, PET‐Aβ load and hippocampal volume in the pre‐clinical stage of AD. Moreover, the associated ACs potentially classified or assisted in categorising the brain Aβ status in CN older adults; therefore, they can be potential predictive markers for initial neuropathological alterations in older adults. However, further research in independent cross‐sectional and longitudinal study cohorts, utilising CSF and plasma P‐tau181 in parallel and accounting for highly characterised AD cohorts, is needed to validate the current findings.

## AUTHOR CONTRIBUTIONS

**Tahmida Sharmin:** Conceptualization; methodology; investigation; formal analysis; visualization; writing – original draft; writing – review and editing. **Pratishtha Chatterjee:** Conceptualization; methodology; data curation; funding acquisition; writing – review and editing. **James D. Doecke:** Formal analysis; validation; writing – review and editing. **Nicholas J. Ashton:** Methodology; data curation. **Kevin Huynh:** Writing – review and editing; formal analysis; validation. **Steve Pedrini:** Writing – review and editing. **Hamid R. Sohrabi:** Data curation; methodology; writing – review and editing. **Benjamin Heng:** Writing – review and editing. **Shaun Eslick:** Writing – review and editing. **Henrik Zetterberg:** Funding acquisition; writing – review and editing; data curation. **Kaj Blennow:** Data curation; funding acquisition. **Manohar Garg:** Writing – review and editing; supervision. **Ralph N. Martins:** Conceptualization; funding acquisition; project administration; writing – review and editing; supervision.

## CONFLICT OF INTEREST STATEMENT

All authors confirm that there are no competing financial or non‐financial interests to report concerning the work described in this manuscript. Nicholas J. Ashton has served as a consultant for Quanterix and has given lectures in symposia sponsored by Lilly, Quanterix and Biogen. Henrik Zetterberg has served at scientific advisory boards and/or as a consultant for Abbvie, Acumen, Alector, Alzinova, ALZPath, Amylyx, Annexon, Apellis, Artery Therapeutics, AZTherapies, Cognito Therapeutics, CogRx, Denali, Eisai, LabCorp, Merry Life, Nervgen, Novo Nordisk, Optoceutics, Passage Bio, Pinteon Therapeutics, Prothena, Red Abbey Labs, reMYND, Roche, Samumed, Siemens Healthineers, Triplet Therapeutics and Wave; has given lectures in symposia sponsored by Alzecure, Biogen, Cellectricon, Fujirebio, Lilly, Novo Nordisk and Roche; and is a co‐founder of Brain Biomarker Solutions in Gothenburg AB (BBS), which is a part of the GU Ventures Incubator Program (outside submitted work). Kaj Blennow has served as a consultant on advisory boards or on data monitoring committees for Acumen, Abcam, ALZpath, AriBio, Axon, BioArctic, Biogen, Eisai, JOMDD/Shimadzu, Julius Clinical, Lilly, MagQu, Novartis, Ono Pharma, Pharmatrophix, Prothena, Roche Diagnostics and Siemens Healthineers, and is a co‐founder of Brain Biomarker Solutions in Gothenburg AB (BBS), which is a part of the GU Ventures Incubator Program (outside the work presented in this paper).

### PEER REVIEW

The peer review history for this article is available at https://www.webofscience.com/api/gateway/wos/peer‐review/10.1111/jnc.16244.

## Supporting information


Data S1.

## Data Availability

The current study database (utilised and/or analysed) will be available at reasonable request from the corresponding author. However, data will not be publicly available because of privacy and ethical restrictions.
