# Supplementary material for: Circulating medium‐ and long‐chain acylcarnitines are associated with plasma P‐tau181 in cognitively normal older adults
Source: J Neurochem. 2024 Oct 30;169(2):10.1111/jnc.16244. doi: 10.1111/jnc.16244 (PMC11808462; doi:10.1111/jnc.16244)
Supplement: Supplementary file 1 — Data S1. [file JNC-169-0-s001.pdf]

Circulating medium and long-chain acylcarnitines are associated with plasma P-tau181 in cognitively normal older adults

Tahmida Sharmin[1, 2], Pratishtha Chatterjee[1, 3], James D. Doecke[4, 5], Nicholas J. Ashton[6, 7], Kevin Huynh[8], Steve Pedrini[5, 9], Hamid R. Sohrabi[1, 9, 10], Benjamin Heng[1], Shaun Eslick[1], Henrik Zetterberg[6, 11, 12, 13, 14, 15], Kaj Blennow[6, 11], Manohar Garg[1], Ralph N. Martins[1, 5, 9]

[1] Macquarie Medical School, Macquarie University, North Ryde, New South Wales, Australia
[2] Department of Pharmacy, University of Rajshahi, Rajshahi, Bangladesh
[3] Florey Institute of Neuroscience and Mental Health, University of Melbourne, Victoria, Australia
[4] Australian eHealth Research Centre, CSIRO, Brisbane, Queensland, Australia
[5] School of Medical and Health Sciences, Edith Cowan University, Perth, Western Australia, Australia
[6] Department of Psychiatry and Neurochemistry, University of Gothenburg, Gothenburg, Sweden
[7] Department of Old Age Psychiatry, Institute of Psychiatry, Psychology & Neuroscience, King's College London, London, UK
[8] Metabolomics Laboratory, Baker Heart and Diabetes Institute, Melbourne, Victoria, Australia
[9] Alzheimer's Research Australia, Western Australia, Australia
[10] School of Psychology, Murdoch University, Western Australia, Australia
[11] Clinical Neurochemistry Laboratory, Sahlgrenska University Hospital, Gothenburg, Sweden
[12] Department of Neurodegenerative Disease, UCL Institute of Neurology, Queen Square, London, UK
[13] UK Dementia Research Institute at UCL, London, UK
[14] Hong Kong Center for Neurodegenerative Diseases, Clear Water Bay, Hong Kong, China
[15] Wisconsin Alzheimer's Disease Research Center, University of Wisconsin School of Medicine and Public Health, University of Wisconsin-Madison, Madison, WI, USA

Correspondence

Professor Ralph N. Martins, Macquarie Medical School, Macquarie University, North Ryde, NSW 2109, Australia. Email: ralph.martins@mq.edu.au, Work phone: +61-2-9850-4573.

Supplementary materials

Supplementary Table 1: Cognitive measures and associated neuropsychological tests.

Cognitive measures	Analysis method	Neuropsychological tests employed
Verbal and visual episodic memory (z scores)	Computed from the mean of the z-score measures	a) Rey Auditory Verbal Learning Test—RAVLT List A, RAVLT short delay & RAVLT long delay (Estévez-González et al., 2003), b) Logical Memory— LM I & LM II (WMS-III; Story A only) (Elwood, 1991), and c) Rey Complex Figure Test—RCFT 3 min & RCFT 30 min (Meyers and Meyers, 1995)

Working memory and executive function (z scores)	Computed from the mean of the z-score measures	a) WAIS-III Digit Span backward (Wechsler, 1987), b) WAIS-III Digit Symbol Substitution Test (DSST) (Wechsler, 1997), and c) D-KEFS: Category Fluency (Boys Names) & Category Switching (Fruits and Furniture) Tasks (Delis et al., 2001)
The global composite (z scores)	Computed from the mean of the z-score measures	a) MMSE (Folstein et al., 1975), b) the verbal and visual episodic memory, & c) the working memory and executive function

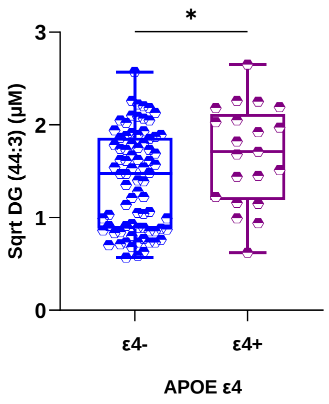

Supplementary Figure 1: Comparison of plasma DG(44:3) between the *APOE* ε4- (n=79) and *APOE* ε4+ (n=21) individuals. Between-group difference was computed employing the independent sample t-test, and the p-value was calculated following square root transformation to meet the requirement for normal distribution. In this box and whisker plot, each box comprises a horizontal bar (reflecting median) and Q3 to Q1 (reflecting interquartile range), and the whiskers indicate the maximum (upper) and the minimum (lower) values.

Supplementary Table 2: The list of plasma metabolites correlated with age in the entire cohort.

Plasma metabolites (µM)	Age	
	r	p-value
**Acylcarnitines**		
AC(3:0) [†]	0.205	0.040
AC(8:1) [†]	0.343	0.000472
AC(10:0) [$]	0.222	0.026
AC(10:2) [$]	0.324	0.001
AC(10:3) [$]	0.320	0.001
AC(12:0) [$]	0.258	0.009
AC(12:1) [$]	0.228	0.023
AC(14:1) [$]	0.225	0.024
**Lysophosphatidylcholines**		
LPC(17:1)	0.232	0.020
**Phosphatidylcholines**		

PC(32:2) †	-0.243	0.015
PC(34:5) $	-0.210	0.036
PC(35:5) †	-0.237	0.018
PC(36:2)	-0.275	0.006
PC(36:5) $	-0.220	0.028
PC(38:2) †	-0.223	0.25
PC(38:5)	-0.216	0.031
PC(40:6) †	-0.227	0.023
PC(40:9) $	-0.232	0.020
PC(42:10) †	-0.204	0.042
PC-O(38:6) $	-0.233	0.020
PC-O(40:6)	-0.237	0.018
PC-O(40:7)	-0.221	0.027
**Sphingomyelins**		
SM(33:1)	-0.208	0.038
SM(38:1) $	-0.204	0.042
SM(39:1) $	-0.222	0.027
SM(40:2)	-0.260	0.009
SM(40:4) †	-0.267	0.007
SM(41:1) $	-0.302	0.002
SM(42:1) $	-0.275	0.006
**Cholesteryl esters**		
CE(18:2)	-0.231	0.021
**Triglycerides**		
TG(56:7) $	-0.272	0.006
**Amino acids**		
Citrulline $	0.216	0.031
Proline $	0.262	0.009
Tryptophan	-0.226	0.024
**Biogenic amines**		
Creatinine $	0.347	0.000399
Met-SO $	0.199	0.048
SDMA $	0.349	0.000366

Correlations were calculated using Pearson correlation. 'r' represents the Pearson correlation coefficient, and p-values < 0.05 were considered significant. † represents square root transformation, and $ represents logarithmic transformation to meet the requirement for normal distribution. Met-SO: Methionine sulfoxide, SDMA: symmetric dimethylarginine.

Supplementary Table 3: The list of plasma metabolites associated with gender in the entire cohort.

Plasma metabolites (µM)	N (M, F)	Male	Female	p-value
**Acylcarnitines**				
AC(3:0) †	32, 68	0.527 ± 0.235	0.390 ± 0.157	0.001
AC(10:3) $	32, 68	0.073 ± 0.040	0.056 ± 0.028	0.033
AC(16:0) †	32, 67	0.099 ± 0.030	0.085 ± 0.025	0.015

AC(18:0) $	32, 68	0.041 ± 0.026	0.031 ± 0.017	0.028
AC(18:1) †	32, 68	0.160 ± 0.045	0.131 ± 0.041	0.002
AC(18:2) †	32, 68	0.076 ± 0.020	0.062 ± 0.018	0.001
**Lysophosphatidylcholines**				
LPC(17:1)	32, 68	0.232 ± 0.060	0.199 ± 0.056	0.008
LPC(18:2)	32, 68	28.891 ± 8.513	25.400 ± 7.880	0.047
LPC(20:4)	32, 68	19.234 ± 5.432	16.721 ± 5.049	0.026
LPC-O(18:1) $	32, 68	0.540 ± 0.151	0.480 ± 0.110	0.042
**Phosphatidylcholines**				
PC(32:2) †	32, 68	4.205 ± 1.972	5.330 ± 2.082	0.008
PC(32:3) †	32, 68	0.989 ± 0.411	1.404 ± 0.565	< 0.001
PC(32:6)	32, 68	0.469 ± 0.202	0.629 ± 0.279	0.005
PC(33:3) †	32, 68	2.053 ± 0.807	2.668 ± 1.183	0.014
PC(33:4)	32, 68	1.044 ± 0.400	1.352 ± 0.485	0.002
PC(34:3)	32, 68	16.258 ± 4.341	19.840 ± 7.019	0.009
PC(34:4) $	32, 68	5.635 ± 2.244	7.545 ± 3.873	0.018
PC(34:5) $	32, 68	1.375 ± 0.601	1.960 ± 0.969	0.003
PC(35:5) †	32, 68	1.067 ± 0.394	1.413 ± 0.605	0.005
PC(36:2)	32, 68	172.791 ± 55.123	201.106 ± 60.499	0.027
PC(36:5) $	32, 68	112.334 ± 37.145	141.696 ± 55.512	0.021
PC(36:6)	32, 68	3.850 ± 1.459	5.320 ± 2.310	0.001
PC(37:5)	32, 68	6.302 ± 2.691	8.087 ± 3.472	0.012
PC(37:6)	32, 68	1.987 ± 0.925	2.547 ± 1.071	0.013
PC(38:2) †	32, 68	3.039 ± 1.544	4.557 ± 2.409	0.003
PC(38:5)	32, 68	94.678 ± 26.500	116.616 ± 37.755	0.004
PC(38:6) $	32, 68	88.934 ± 24.851	111.866 ± 34.753	0.002
PC(39:6)	32, 68	2.696 ± 0.995	3.332 ± 1.205	0.011
PC(39:7)	32, 68	2.399 ± 1.164	2.906 ± 1.197	0.049
PC(40:3)	32, 68	0.965 ± 0.335	1.141 ± 0.433	0.046
PC(40:5)	32, 68	10.679 ± 2.531	12.702 ± 3.750	0.007
PC(40:6) †	32, 68	30.697 ± 8.586	39.725 ± 12.409	< 0.001
PC(40:8) †	32, 68	17.199 ± 6.000	21.766 ± 8.834	0.014
PC(40:9) $	32, 68	20.467 ± 8.599	29.279 ± 12.950	0.001
PC(41:4)	32, 68	1.182 ± 0.459	1.601 ± 0.632	0.001
PC(41:5)	32, 68	0.664 ± 0.277	0.810 ± 0.255	0.011
PC(41:8) †	32, 68	0.374 ± 0.164	0.487 ± 0.224	0.017
PC(42:10) †	32, 68	1.692 ± 0.744	2.477 ± 1.085	< 0.001
PC-O(32:1) †	32, 68	1.099 ± 0.451	1.351 ± 0.459	0.011
PC-O(34:4)	32, 68	0.683 ± 0.319	0.877 ± 0.365	0.012
PC-O(36:6) $	32, 68	2.419 ± 0.940	3.184 ± 1.467	0.009
PC-O(37:6) †	32, 68	0.463 ± 0.164	0.581 ± 0.226	0.009
PC-O(38:6) $	32, 68	6.595 ± 2.047	8.287 ± 2.652	0.002
PC-O(40:6)	32, 68	2.779 ± 0.862	3.195 ± 0.904	0.032
PC-O(40:7)	32, 68	3.866 ± 1.200	4.692 ± 1.586	0.010
**Sphingomyelins**				
SM(30:1) $	32, 68	0.371 ± 0.107	0.556 ± 0.220	< 0.00001

SM(32:1) †	32, 68	8.339 ± 1.757	10.778 ± 2.902	< 0.0001
SM(32:2)	32, 68	0.642 ± 0.210	1.088 ± 0.339	< 0.000000001
SM(33:1)	32, 68	25.652 ± 11.772	35.423 ± 15.287	0.002
SM(34:1) †	32, 68	83.253 ± 19.489	97.429 ± 22.118	0.003
SM(34:2)	32, 68	11.592 ± 2.940	15.184 ± 3.537	< 0.00001
SM(35:1)	32, 68	2.769 ± 0.626	3.314 ± 0.922	0.003
SM(36:2)	32, 68	4.593 ± 1.333	6.179 ± 1.613	< 0.00001
SM(38:1) $	32, 68	21.027 ± 8.985	23.551 ± 6.368	0.023
SM(38:2)	32, 68	8.307 ± 3.191	9.997 ± 2.753	0.008
SM(39:1) $	32, 68	6.895 ± 2.671	8.307 ± 2.429	0.002
SM(40:2)	32, 68	21.816 ± 7.780	27.375 ± 6.392	0.001
SM(40:4) †	32, 68	4.450 ± 1.423	6.063 ± 2.146	< 0.001
SM(41:1) $	32, 68	12.793 ± 4.050	14.725 ± 4.232	0.023
SM(41:2)	32, 68	11.264 ± 2.594	14.683 ± 3.846	< 0.0001
SM(42:2)	32, 68	52.259 ± 16.502	59.124 ±14.056	0.034
SM(42:3)	32, 68	23.158 ± 8.479	28.687 ± 7.773	0.002
**Cholesteryl esters**				
CE(16:0)	32, 68	177.650 ± 64.104	229.400 ± 89.376	0.004
CE(16:1)	32, 68	142.199 ± 62.057	215.853 ± 92.128	< 0.0001
CE(17:1)	32, 68	14.148 ± 5.428	17.266 ± 6.147	0.016
CE(18:1)	32, 68	1080.65 ± 380.60	1303.13 ± 400.96	0.010
CE(18:2)	32, 68	3674.37 ± 1406.93	4716.59 ± 1502.96	0.001
CE(18:3)	32, 68	180.819 ± 65.825	238.525 ± 99.095	0.003
CE(20:5) $	32, 68	294.563 ± 201.602	419.338 ± 313.880	0.002
CE(22:6) $	32, 68	151.472 ± 61.235	206.116 ± 73.011	< 0.0001
**Diglycerides**				
DG(41:1)	32, 68	7.360 ± 2.740	8.947 ± 2.808	0.007
DG-O(36:4)	32, 68	1.637 ± 0.378	1.935 ± 0.478	0.003
**Triglycerides**				
TG(54:2)	32, 68	33.170 ± 21.759	25.413 ± 15.312	0.043
**Amino acids**				
Aspartate $	32, 68	4.5 ± 0.827	4.142 ± 0.654	0.028
Glutamate $	32, 68	51.33 ± 21.55	41.79 ± 16.97	0.024
Glycine $	32, 68	201.93 ± 46.19	246.76 ± 66.87	< 0.001
Isoleucine	32, 68	75.48 ± 13.76	63.30 ± 11.41	< 0.0001
Leucine + Isoleucine	32, 68	168.27 ± 33.06	140.17 ± 24.84	< 0.00001
Methionine	32, 68	22.23 ± 3.19	20.27 ± 3.37	0.007
Proline $	32, 68	196.17 ± 75.26	167.79 ± 49.41	0.029
Valine	32, 68	218.65 ± 39.91	195.66 ± 39.53	0.008
**Biogenic amines**				
Creatinine $	32, 68	86.53 ± 28.22	63.94 ± 16.61	< 0.00001

p-values were calculated via the Independent sample t-test, and p-values < 0.05 were considered statistically significant. † represents square root transformation, and $ represents logarithmic transformation to meet the requirement for normal distribution.

Supplementary Table 4: The list of plasma metabolites correlated with Body Mass Index (BMI) in the entire cohort.

Plasma metabolites (µM)	BMI	
	r	p-value
**Phosphatidylcholines**		
PC(39:5)	-0.209	0.037
PC(42:6) †	-0.203	0.043
PC(44:1) †	0.234	0.020
PC-O(36:2) †	-0.204	0.042
**Sphingomyelins**		
SM(36:0) †	0.315	0.001
SM(36:2)	0.205	0.041
SM(44:1) $	0.247	0.013
**Carbohydrates and related**		
Hexoses $	0.255	0.010
**Cholesteryl esters**		
CE(16:1)	0.205	0.041
CE(18:3)	0.221	0.027
CE(19:2)	-0.317	0.001
**Diglycerides**		
DG(34:1) $	0.343	< 0.001
DG(36:2) †	0.281	0.005
DG(38:5) $	0.243	0.015
DG-O(32:2)	0.337	0.001
DG-O(34:1)	0.335	0.001
**Triglycerides**		
TG(50:1) †	0.287	0.004
TG(50:2) †	0.303	0.002
TG(50:3) $	0.248	0.013
TG(52:2) †	0.288	0.004
TG(54:2)	0.207	0.039
**Amino acids**		
Alanine	0.343	< 0.001
Glutamate $	0.380	< 0.0001
Proline $	0.262	0.008
Tyrosine	0.255	0.011
Valine	0.226	0.024
**Biogenic amines**		
Kynurenine $	0.291	0.003
ADMA	0.222	0.027

Correlations were calculated using Pearson correlation. 'r' represents the Pearson correlation coefficient, and p-values < 0.05 were considered significant. † represents square root transformation, and $ represents logarithmic transformation to meet the requirement for normal distribution. ADMA: asymmetric dimethylarginine.

Supplementary Table 5: Associations of verbal and visual episodic memory with P-tau181-linked metabolites in all participants, CN Aβ- and CN Aβ+

	All participants (n=100)				CN Aβ- (n=65)				CN Aβ+ (n=35)			
	**ρ**	**p-value**	**β**	**p-value**	**ρ**	**p-value**	**β**	**p-value**	**ρ**	**p-value**	**β**	**p-value**
**Lipids**												
AC(8:1) †	-0.100	.322	-0.402	.520	-0.057	.651	0.030	.964	-0.137	.432	-1.235	.296
AC(10:0) $	-0.268	**.007**	-0.505	**.034**	-0.171	.174	-0.157	.546	-0.430	**.010**	-1.222	**.005** ‡
AC(10:1)	-0.192	.056	-1.788	.125	-0.126	.317	-0.251	.857	-0.308	.072	-4.043	**.029**
AC(10:2) $	-0.164	.104	-0.120	.469	-0.107	.398	-0.032	.855	-0.328	.054	-0.595	.072
AC(10:3) $	-0.204	**.042**	-0.263	.372	-0.162	.317	0.071	.830	-0.388	**.021**	-1.291	**.008** ‡
AC(12:0) $	-0.292	**.003**	-0.415	.113	-0.229	.066	-0.174	.607	-0.372	**.027**	-0.520	.195
AC(12:1) $	-0.295	**.003**	-0.702	**.018** ‡	-0.174	.166	-0.119	.719	-0.474	**.004**	-1.837	**.001** ‡
AC(13:0) $	-0.094	.354	-0.159	.460	-0.082	.519	-0.046	.840	-0.112	.523	-0.396	.382
AC(14:1) $	-0.285	**.004**	-0.526	**.015** ‡	-0.216	.085	-0.323	.183	-0.407	**.015**	-1.227	**.002** ‡
AC(16:0) †	-0.255	**.011**	-3.325	**.021**	-0.192	.128	-2.084	.196	0.334	.050	-6.616	**.003** ‡
AC(18:1) †	-0.348	**.0003**	-3.266	**.002** ‡	-0.326	**.008**	-2.530	**.034**	-0.349	**.039**	-4.984	**.005** ‡
LPC(17:1)	-0.215	**.032**	-1.226	.274	-0.218	.080	-1.308	.309	-0.215	.215	-0.848	.677
TG(56:7) $	0.081	.425	-0.262	.442	0.118	.349	0.238	.524	0.004	.982	-1.159	.077
**Amino acids**												
Citrulline $	-0.051	.612	-0.184	.717	0.061	.631	0.683	.290	-0.246	.154	-1.049	.142
Proline $	-0.171	.090	-0.230	.642	-0.139	.270	-0.358	.570	-0.182	.295	-0.389	.616
**Biogenic amines**												
Creatinine $	-0.259	**.009**	-0.772	.184	-0.208	.096	-0.498	.478	-0.331	.052	-1.591	.102
Kynurenine $	-0.008	.933	-0.037	.944	-0.005	.971	0.242	.672	0.038	.830	-0.853	.417
SDMA $	-0.140	.165	-0.387	.405	-0.151	.230	-0.424	.475	-0.103	.556	-0.495	.594

Correlations of verbal and visual episodic memory with plasma metabolites linked with P-tau181 were calculated using the Spearman correlation, and p-values < 0.05 were considered significant. Associations of verbal and visual episodic memory with plasma metabolites linked with P-tau181 upon adjusting for age, sex, APOE ε4 status and BMI were calculated using the generalised linear model and p-values < 0.05 that survived FDR adjustment (denoted as ‡) were considered significant. 'n' represents the number of participants, 'ρ' represents the Spearman correlation coefficient and 'β' represents the beta coefficient. † represents square root transformation, and $ represents logarithmic transformation to meet the requirement for normal distribution. AC: acylcarnitine, LPC: lysophosphatidylcholine, TG: triglyceride, SDMA: symmetric dimethylarginine. P-tau181-linked metabolites significantly correlating (p-values < 0.05, **bold**) with verbal and visual episodic memory both before and after adjusting for confounding variables and survived FDA correction (p-values < 0.05, **bold** and "‡" signed) were considered statistically significant.

Supplementary Table 6: Associations of working memory and executive function with P-tau181-linked metabolites in all participants, CN Aβ- and CN Aβ+

	All participants (n=100)				CN Aβ- (n=65)				CN Aβ+ (n=35)			
	ρ	p-value	β	p-value	ρ	p-value	β	p-value	ρ	p-value	β	p-value
**Lipids**												
AC(8:1) †	-0.152	.132	-0.196	.740	-0.110	.382	0.070	.922	-0.200	.249	0.188	.850
AC(10:0) $^{\$}$	0.090	.373	0.475	**.034**	0.128	.310	0.532	**.047**	0.111	.524	0.493	.209
AC(10:1)	0.045	.659	1.550	.160	0.034	.789	1.282	.379	0.154	.379	2.126	.182
AC(10:2) $^{\$}$	-0.210	**.036**	-0.143	.360	-0.222	.076	-0.131	.475	-0.171	.327	0.020	.944
AC(10:3) $^{\$}$	-0.015	.880	0.366	.187	0.039	.758	0.557	.107	-0.116	.508	0.264	.549
AC(12:0) $^{\$}$	-0.022	.826	0.283	.257	-0.004	.973	0.455	.198	0.029	.867	0.199	.557
AC(12:1) $^{\$}$	-0.016	.875	0.278	.334	0.020	.873	0.423	.222	0.002	.993	0.207	.689
AC(13:0) $^{\$}$	-0.070	.492	0.017	.935	-0.155	.217	-0.080	.738	0.107	.539	0.543	.141
AC(14:1) $^{\$}$	0.008	.940	0.225	.283	0.070	.577	0.280	.276	-0.036	.836	-0.011	.976
AC(16:0) †	0.005	.959	1.086	.423	0.029	.821	0.978	.567	0.087	.620	0.439	.829
AC(18:1) †	0.024	.810	0.871	.404	0.046	.715	1.027	.429	0.120	.493	0.492	.761
LPC(17:1)	-0.081	.420	-0.083	.938	0.132	.293	2.013	.134	-0.292	.089	-1.583	.343
TG(56:7) $^{\$}$	-0.007	.946	-0.322	.317	0.154	.219	0.430	.272	-0.286	.096	-1.659	**.001** ‡
**Amino acids**												
Citrulline $^{\$}$	-0.103	.306	-0.155	.747	0.035	.783	0.943	.165	-0.308	.072	-1.133	.050
Proline $^{\$}$	-0.151	.132	-0.064	.892	-0.101	.423	0.138	.836	-0.198	.254	0.220	.733
**Biogenic amines**												
Creatinine $^{\$}$	-0.216	**.031**	-0.473	.391	-0.083	.509	0.471	.525	-0.326	.056	-1.244	.125
Kynurenine $^{\$}$	-0.130	.196	-0.199	.691	0.024	.846	0.755	.207	-0.308	.072	-1.679	**.044**
SDMA $^{\$}$	-0.179	.075	-0.209	.674	-0.104	.409	0.033	.958	-0.253	.142	-0.083	.915

Correlations of working memory and executive function with plasma metabolites linked with P-tau181 were calculated using the Spearman correlation, and p-values < 0.05 were considered significant. Associations of verbal and visual episodic memory with plasma metabolites linked with P-tau181 upon adjusting for age, sex, APOE ε4 status and BMI were calculated using the generalised linear model and p-values < 0.05 that survived FDR adjustment (denoted as ‡) were considered significant. 'n' represents the number of participants, 'ρ' represents the Spearman correlation coefficient and 'β' represents the beta coefficient. † represents square root transformation, and $^{\$}$ represents logarithmic transformation to meet the requirement for normal distribution. AC: acylcarnitine, LPC: lysophosphatidylcholine, TG: triglyceride, SDMA: symmetric dimethylarginine. P-tau181-linked metabolites significantly correlating (p-values < 0.05, **bold**) with working memory and executive function both before and after adjusting for confounding variables and survived FDA correction (p-values < 0.05, **bold** and "‡" signed) were considered statistically significant.

Supplementary Table 7: Associations of the Global composite score with P-tau181-linked metabolites in all participants, CN Aβ- and CN Aβ+

	**All participants (n=100)**				**CN Aβ- (n=65)**				**CN Aβ+ (n=35)**			
	**ρ**	**p-value**	**β**	**p-value**	**ρ**	**p-value**	**β**	**p-value**	**ρ**	**p-value**	**β**	**p-value**
**Lipids**												
AC(8:1) [†]	-0.180	.073	-0.348	.459	-0.128	.308	0.058	.911	-0.211	.223	-0.859	.330
AC(10:0) [$]	-0.205	**.041**	-0.178	.327	-0.113	.372	0.065	.749	-0.379	**.025**	-0.683	**.046**
AC(10:1)	-0.178	.077	-0.729	.409	-0.156	.215	0.165	.878	-0.257	.136	-2.123	.136
AC(10:2) [$]	-0.279	**.005**	-0.151	.222	-0.270	**.030**	-0.080	.553	-0.378	**.025**	-0.412	.097
AC(10:3) [$]	-0.216	**.031**	-0.063	.776	-0.130	.304	0.223	.382	-0.419	**.012**	-0.722	.057
AC(12:0) [$]	-0.279	**.005**	-0.204	.304	-0.227	.070	0.0001	1.00	-0.348	**.040**	-0.344	.252
AC(12:1) [$]	-0.287	**.004**	-0.377	.097	-0.200	.110	0.031	.903	-0.418	**.013**	-1.179	**.005** ‡
AC(13:0) [$]	-0.123	.224	-0.101	.532	-0.149	.236	-0.066	.708	-0.086	.624	-0.084	.805
AC(14:1) [$]	-0.276	**.005**	-0.303	.067	-0.217	.082	-0.160	.397	-0.397	**.018**	-0.854	**.005** ‡
AC(16:0) [†]	-0.217	**.031**	-1.838	.084	-0.133	.296	-0.977	.436	-0.338	**.047**	-4.402	**.009** ‡
AC(18:1) [†]	-0.281	**.005**	-1.853	**.022**	-0.250	**.045**	-1.264	.180	-0.292	.089	-3.180	**.019** ‡
LPC(17:1)	-0.215	**.032**	-0.803	.342	-0.121	.335	0.188	.243	-0.292	.089	-0.250	.351
TG(56:7) [$]	0.107	.290	-0.211	.410	-0.266	**.032**	0.398	.164	-0.128	.462	-1.311	**.004** ‡
**Amino acids**												
Citrulline [$]	-0.132	.192	-0.195	.610	-0.031	.806	0.638	.201	-0.330	.053	-0.992	.057
Proline [$]	-0.198	**.048**	-0.158	.671	-0.129	.304	-0.088	.857	-0.251	.147	-0.200	.729
**Biogenic amines**												
Creatinine [$]	-0.328	**.001**	-0.685	.115	-0.246	**.049**	-0.224	.681	-0.398	**.018**	-1.429	**.045**
Kynurenine [$]	-0.096	.341	-0.155	.696	-0.063	.616	0.314	.478	-0.117	.504	-1.123	.143
SDMA [$]	-0.238	**.017**	-0.456	.245	-0.267	**.031**	-0.436	.340	-0.196	.260	-0.358	.605

Correlations of the Global composite score with plasma metabolites linked with P-tau181 were calculated using the Spearman correlation, and p-values < 0.05 were considered significant. Associations of verbal and visual episodic memory with plasma metabolites linked with P-tau181 upon adjusting for age, sex, APOE ε4 status and BMI were calculated using the generalised linear model and p-values < 0.05 that survived FDR adjustment (denoted as ‡) were considered significant. 'n' represents the number of participants, 'ρ' represents the Spearman correlation coefficient and 'β' represents the beta coefficient. [†] represents square root transformation, and [$] represents logarithmic transformation to meet the requirement for normal distribution. AC: acylcarnitine, LPC: lysophosphatidylcholine, TG: triglyceride, SDMA: symmetric dimethylarginine. P-tau181-linked metabolites significantly correlating (p-values < 0.05, **bold**) with the Global composite score both before and after adjusting for confounding variables and survived FDA correction (p-values < 0.05, **bold** and "‡" signed) were considered statistically significant.

Supplementary Table 8: Associations of PET-Aβ load with P-tau181-linked metabolites in all participants, CN Aβ- and CN Aβ+

	All participants (n=100)				CN Aβ- (n=65)				CN Aβ+ (n=35)			
	ρ	p-value	β	p-value	ρ	p-value	β	p-value	ρ	p-value	β	p-value
**Lipids**												
AC(8:1) †	0.121	.232	0.322	.228	0.109	.388	0.057	.569	0.065	.712	0.180	.687
AC(10:0) $	0.137	.175	0.226	**.027**	-0.055	.665	-0.031	.415	0.348	**.041**	0.463	**.004** ‡
AC(10:1)	0.070	.488	0.832	.096	0.014	.914	-0.203	.322	0.118	.501	1.262	.073
AC(10:2) $	0.159	.115	0.063	.371	0.301	**.015**	0.052	**.039**	0.023	.894	-0.045	.729
AC(10:3) $	0.036	.719	0.072	.570	0.065	.605	-0.020	.682	0.340	**.045**	0.387	**.039**
AC(12:0) $	0.164	.103	0.196	.081	0.028	.822	-0.039	.435	0.212	.221	0.181	.226
AC(12:1) $	0.140	.164	0.237	.066	0.099	.433	0.002	.973	0.183	.292	0.350	.120
AC(13:0) $	0.119	.236	0.149	.104	0.047	.711	-0.010	.766	0.289	.092	0.340	**.034**
AC(14:1) $	0.057	.574	0.119	.211	-0.094	.455	-0.053	.137	0.310	.070	0.393	**.010**
AC(16:0) †	0.113	.266	0.474	.436	-0.002	.985	-0.287	.231	0.356	**.035**	2.318	**.005** ‡
AC(18:1) †	0.083	.410	0.707	.132	-0.030	.814	-0.159	.383	0.271	.116	1.805	**.006** ‡
LPC(17:1)	0.247	**.013**	0.767	.109	0.098	.437	0.046	.810	0.316	.064	0.615	.414
TG(56:7) $	-0.039	.703	-0.045	.761	0.095	.453	0.080	.146	-0.010	.954	-0.145	.568
**Amino acids**												
Citrulline $	0.039	.702	-0.022	.920	0.024	.849	-0.043	.656	0.086	.622	-0.109	.690
Proline $	0.183	.068	-0.005	.983	0.251	**.044**	0.120	.194	0.071	.685	-0.027	.927
**Biogenic amines**												
Creatinine $	0.190	.058	0.169	.501	0.121	.335	-0.054	.607	0.096	.585	0.117	.755
Kynurenine $	0.144	.153	0.120	.598	0.075	.553	-0.011	.895	-0.042	.809	-0.252	.521
SDMA $	0.128	.206	0.166	.460	0.009	.943	-0.071	.420	0.074	.674	0.149	.667

Correlations of PET-Aβ load with plasma metabolites linked with P-tau181 were calculated using the Spearman correlation, and p-values < 0.05 were considered significant. Associations of verbal and visual episodic memory with plasma metabolites linked with P-tau181 upon adjusting for age, sex, APOE ε4 status and BMI were calculated using the generalised linear model and p-values < 0.05 that survived FDR adjustment (denoted as ‡) were considered significant. 'n' represents the number of participants, 'ρ' represents the Spearman correlation coefficient and 'β' represents the beta coefficient. † represents square root transformation, and $ represents logarithmic transformation to meet the requirement for normal distribution. AC: acylcarnitine, LPC: lysophosphatidylcholine, TG: triglyceride, SDMA: symmetric dimethylarginine. P-tau181-linked metabolites significantly correlating (p-values < 0.05, **bold**) with PET-Aβ load both before and after adjusting for confounding variables and survived FDA correction (p-values < 0.05, **bold** and "‡" signed) were considered statistically significant.

Supplementary Table 9: Associations of hippocampal volume left with P-tau181-linked metabolites in all participants, CN Aβ- and CN Aβ+

	All participants (n=96)				CN Aβ- (n=64)				CN Aβ+ (n=32)			
	ρ	p-value	β	p-value	ρ	p-value	β	p-value	ρ	p-value	β	p-value
**Lipids**												
AC(8:1) †	-0.247	**.015**	-0.039	**.039**	-0.255	**.042**	-0.034	.149	-0.246	.175	-0.058	**.042**
AC(10:0) $	-0.147	.153	-0.008	.300	-0.124	.330	-0.006	.532	-0.201	.271	-0.017	.155
AC(10:1)	-0.086	.403	-0.011	.752	-0.027	.831	0.004	.928	-0.219	.228	-0.072	.130
AC(10:2) $	-0.207	**.043**	-0.007	.182	-0.193	.127	-0.006	.354	-0.274	.129	-0.025	**.006** ‡
AC(10:3) $	-0.243	**.017**	-0.016	.072	-0.295	**.018**	-0.024	**.036**	-0.157	.391	-0.009	.488
AC(12:0) $	-0.120	.245	-0.002	.801	-0.127	.317	-0.003	.799	-0.120	.541	-0.009	.480
AC(12:1) $	-0.143	.164	-0.010	.285	-0.114	.368	-0.006	.630	-0.180	.326	-0.029	.067
AC(13:0) $	-0.082	.425	-0.002	.771	-0.018	.888	0.003	.691	-0.199	.276	-0.018	.096
AC(14:1) $	-0.119	.247	-0.0003	.953	-0.078	.538	0.002	.779	-0.209	.251	-0.022	**.044**
AC(16:0) †	-0.046	.659	0.020	.644	0.049	.706	0.058	.309	-0.245	.176	-0.094	.120
AC(18:1) †	-0.180	.079	-0.026	.448	-0.195	.123	-0.027	.532	-0.180	.324	-0.044	.395
LPC(17:1)	-0.167	.103	-0.018	.615	-0.097	.444	-0.006	.895	-0.317	.078	-0.044	.388
TG(56:7) $	-0.012	.909	-0.008	.424	0.003	.978	0.002	.864	-0.068	.713	-0.023	.168
**Amino acids**												
Citrulline $	-0.099	.338	-0.003	.836	-0.072	.572	-0.011	.655	-0.151	.410	0.003	.875
Proline $	-0.074	.476	-0.002	.881	-0.036	.776	0.003	.892	-0.161	.379	-0.005	.822
**Biogenic amines**												
Creatinine $	-0.182	.076	-0.014	.424	-0.188	.138	-0.027	.283	-0.129	.482	-0.011	.731
Kynurenine $	-0.104	.311	-0.013	.427	-0.143	.259	-0.018	.394	0.036	.846	-0.018	.485
SDMA $	-0.142	.169	-0.009	.587	-0.211	.094	-0.026	.219	0.031	.867	0.015	.527

Correlations of hippocampal volume left with plasma metabolites linked with P-tau181 were calculated using the Spearman correlation, and p-values < 0.05 were considered significant. Associations of verbal and visual episodic memory with plasma metabolites linked with P-tau181 upon adjusting for age, sex, APOE ε4 status and BMI were calculated using the generalised linear model and p-values < 0.05 that survived FDR adjustment (denoted as ‡) were considered significant. 'n' represents the number of participants, 'ρ' represents the Spearman correlation coefficient and 'β' represents the beta coefficient. † represents square root transformation, and $ represents logarithmic transformation to meet the requirement for normal distribution. AC: acylcarnitine, LPC: lysophosphatidylcholine, TG: triglyceride, SDMA: symmetric dimethylarginine. P-tau181-linked metabolites significantly correlating (p-values < 0.05, **bold**) with hippocampal volume left both before and after adjusting for confounding variables and survived FDA correction (p-values < 0.05, **bold** and "‡" signed) were considered statistically significant.

Supplementary Table 10: Associations of hippocampal volume right with P-tau181-linked metabolites in all participants, CN Aβ- and CN Aβ+

	All participants (n=96)				CN Aβ- (n=64)				CN Aβ+ (n=32)			
	ρ	p-value	β	p-value	ρ	p-value	β	p-value	ρ	p-value	β	p-value
**Lipids**												
AC(8:1) †	-0.247	**.015**	-0.037	.054	-0.247	**.049**	-0.028	.244	-0.254	.161	-0.060	**.032**
AC(10:0) $	-0.069	.503	-0.003	.644	0.001	.996	0.005	.620	-0.218	.232	-0.025	**.026** ‡
AC(10:1)	-0.040	.695	0.004	.914	0.052	.684	0.055	.267	-0.210	.249	-0.094	**.044**
AC(10:2) $	-0.189	.066	-0.006	.225	-0.149	.241	-0.004	.549	-0.303	.092	-0.026	**.004** ‡
AC(10:3) $	-0.224	**.028**	-0.013	.144	-0.223	.077	-0.013	.267	-0.212	.243	-0.018	.184
AC(12:0) $	-0.087	.401	0.001	.889	-0.039	.760	0.012	.311	-0.204	.263	-0.018	.116
AC(12:1) $	-0.142	.167	-0.012	.218	-0.097	.446	-0.003	.815	-0.223	.221	-0.035	**.019** ‡
AC(13:0) $	-0.002	.988	-0.002	.719	0.080	.530	0.007	.409	-0.231	.203	-0.023	**.030**
AC(14:1) $	-0.108	.294	-0.005	.460	-0.014	.911	0.003	.706	-0.301	.094	-0.034	**.0004** ‡
AC(16:0) †	-0.094	.363	-0.011	.799	0.041	.752	0.071	.219	-0.383	**.031**	-0.186	**.0004** ‡
AC(18:1) †	-0.243	**.017**	-0.061	.074	-0.243	.053	-0.045	.299	-0.259	.153	-0.105	**.030** ‡
LPC(17:1)	-0.140	.174	-0.031	.373	-0.019	.880	-0.003	.949	-0.426	**.015**	-0.093	.058
TG(56:7) $	-0.043	.674	-0.012	.244	0.067	.600	0.0001	.989	-0.275	.128	-0.025	.125
**Amino acids**												
Citrulline $	-0.093	.367	-0.0003	.981	0.012	.923	0.014	.555	-0.291	.106	-0.015	.405
Proline $	-0.115	.264	-0.005	.756	-0.063	.623	0.012	.591	-0.215	.237	-0.029	.168
**Biogenic amines**												
Creatinine $	-0.168	.102	-0.007	.711	-0.142	.262	0.007	.776	-0.177	.334	-0.028	.252
Kynurenine $	-0.189	.065	-0.018	.284	-0.170	.180	-0.006	.768	-0.173	.345	-0.051	**.040**
SDMA $	-0.171	.097	-0.009	.564	-0.145	.253	-0.0003	.986	-0.176	.335	-0.021	.354

Correlations of hippocampal volume right with plasma metabolites linked with P-tau181 were calculated using the Spearman correlation, and p-values < 0.05 were considered significant. Associations of verbal and visual episodic memory with plasma metabolites linked with P-tau181 upon adjusting for age, sex, APOE ε4 status and BMI were calculated using the generalised linear model and p-values < 0.05 that survived FDR adjustment (denoted as ‡) were considered significant. 'n' represents the number of participants, 'ρ' represents the Spearman correlation coefficient and 'β' represents the beta coefficient. † represents square root transformation, and $ represents logarithmic transformation to meet the requirement for normal distribution. AC: acylcarnitine, LPC: lysophosphatidylcholine, TG: triglyceride, SDMA: symmetric dimethylarginine. P-tau181-linked metabolites significantly correlating (p-values < 0.05, **bold**) with hippocampal volume right both before and after adjusting for confounding variables and survived FDA correction (p-values < 0.05, **bold** and "‡" signed) were considered statistically significant.
